# Computational mass spectrometry and genome mining guided discovery of metallophores produced by Microbulbifer

**DOI:** 10.1093/ismeco/ycag238

**Published:** 2026-08-19

**Authors:** Mónica Monge-Loría, Colin Brady, Hongwei Wu, Allegra Aron, Neha Garg

**Affiliations:** School of Chemistry and Biochemistry, Georgia Institute of Technology, 950 Atlantic Drive, Atlanta, GA 30332, United States; Department of Chemistry and Biochemistry, University of Denver, 2101 E. Wesley Ave, Denver, CO 80210, United States; School of Chemistry and Biochemistry, Georgia Institute of Technology, 950 Atlantic Drive, Atlanta, GA 30332, United States; Department of Chemistry and Biochemistry, University of Denver, 2101 E. Wesley Ave, Denver, CO 80210, United States; School of Chemistry and Biochemistry, Georgia Institute of Technology, 950 Atlantic Drive, Atlanta, GA 30332, United States; Center for Microbial Dynamics and Infection, Georgia Institute of Technology, 315 Ferst Drive, Atlanta, GA 30332, United States

**Keywords:** metabolomics, *Microbulbifer*, bulbichelin, acyl petrobactin, siderophore, MassQL, metal competition, microbial ecology

## Abstract

Iron is an essential component of cellular biology. Thus, iron’s low bioavailability is a key evolutionary pressure guiding microbial dynamics in the marine environment. Among marine bacteria, *Microbulbifer* is a chemically underexplored and functionally versatile bacterial genus, which is commonly associated with sponges, algae, corals, and sediments. Previously, genome analyses have revealed that *Microbulbifer* spp. can degrade polymers and synthesize natural products. Despite their recognized potential to produce secondary metabolites, siderophores are yet to be identified in *Microbulbifer*, and their iron acquisition strategies remain largely unknown. Here, we developed a comprehensive mass spectrometry-based query language code to determine siderophore production by *Microbulbifer* spp. in mono- and mixed cultures. Using this workflow, we discovered a new metallophore, which we named bulbichelin, as well as a suite of previously unreported petrobactins containing an unprecedented longer chain length acylation on the central spermidine moiety. We applied genome mining methods to describe the biosynthesis of these compounds. Using metal infusion mass spectrometry, we show that bulbichelins bind a variety of metals. Notably, neither of these compounds were produced in a co-culture of *Microbulbifer* with coral-derived pathogen *Vibrio coralliilyticus* Cn52-H1. Understanding how siderophores shape interspecies interactions between *Microbulbifer* spp. and other marine organisms will aid in unraveling the chemical and catalytic versatility of this genus and adaptation in nutrient deplete marine environment.

## Graphical Abstract

**Graphical Abstract:**
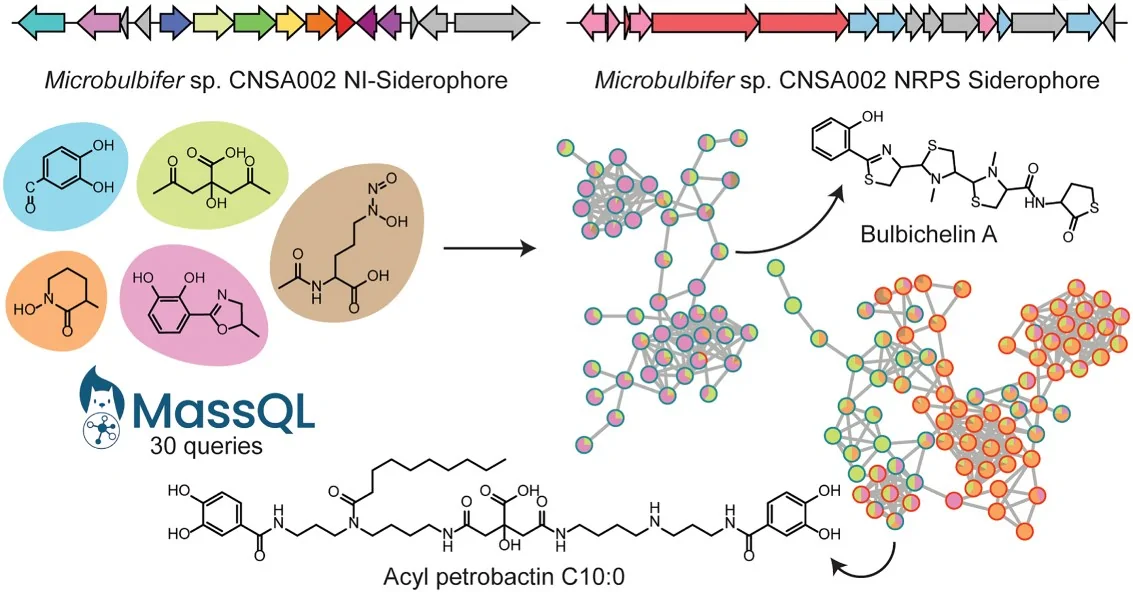

## Introduction

*Microbulbifer* spp. bacteria, common in marine microbiomes, are recognized for their ability to enzymatically degrade biopolymers such as cellulose, chitin, and agar [1–3]. Additionally, multiple biosynthetic gene clusters (BGCs) that encode biosynthesis of specialized metabolites have been identified in their genomes [4, 5]. These analyses have revealed the genetic potential of *Microbulbifer* to produce non-ribosomal peptides (NRPs), polyketides, ribosomally-synthesized and post-translationally modified peptides (RiPPs), ectoine, butyrolactones, polybrominated compounds, and metallophores [4, 6]. A recent genomic inventory of *Microbulbifer* identified 73 gene cluster families [6], however, only a few compounds have been isolated or identified in this genus, with the subset of *Microbulbifer* metabolites consisting of bulbiferates [7], bulbiferamides [8, 9], pseudobulbiferamides [10], and the chalkophore bulbicupramide [6]. Compounds, such as bulbiferates A and B (antibacterial acetamidohydroxybenzoates), are postulated to play key roles in chemical defense and microbial competition [7–9], illustrating their function in ecological interactions. Thus, the disparity between the number of BGCs present in *Microbulbifer* genomes and the number of identified compounds encoded by these BGCs highlights a significant gap. This discrepancy presents an opportunity to explore the ecological roles of these compounds in marine microbiomes and discover novel bioactive molecules [5].

As *Microbulbifer* spp. bacteria are associated with commensal microbiomes, we previously explored production of antimicrobial compounds by *Microbulbifer* against a marine pathogen, *Vibrio coralliilyticus*, isolated from a similar environment [11]. This work led to the discovery of the ability of *Microbulbifer* sp. bacteria to enzymatically degrade amphibactin, an iron-acquiring siderophore produced by the pathogen *V. coralliilyticus*. This mechanism involves proteolytic cleavage of the peptide backbone, which reduces the iron-binding affinity of the cleaved product. This finding suggests that *Microbulbifer* can disrupt the iron acquisition of *V. coralliilyticus*, impacting its survival and proliferation in the host. However, the iron acquisition mechanism of *Microbulbifer* itself remains unknown. Iron is an essential element for almost all living organisms, as it is involved in oxygen transport and storage [12], DNA synthesis and repair [13], cellular respiration [14], and electron transport [15, 16]. In addition to its redox properties, the prevalence of iron as a cofactor reflects iron’s abundance and availability during early life development [17, 18]. Therefore, iron influences microbial ecology by acting as a limiting nutrient, a cofactor for essential enzymes, and a driver of electron transport. Competition for iron therefore became an evolutionary driver for the development of iron acquisition strategies, especially in aquatic environments where bioavailable iron concentration is low, as introduced below.

The iron concentration in the ocean is at sub nanomolar level whereas its biological requirement spans the nanomolar to micromolar range [19, 20]. This discrepancy prompted the “iron hypothesis”, where iron is proposed to be the limiting nutrient for primary producers in the marine environment [21]. Iron limitation has also been shown to induce metabolic alterations in heterotrophic bacteria, primarily in respiration and carbon metabolism [22]. In order to acquire this essential trace metal, bacteria can utilize both ferrous (Fe(II)) and ferric (Fe(III)) iron uptake mechanisms. Ferrous iron uptake systems (i.e. Feo) are limited in marine systems, as this metal species is prevalent only in highly acidic, reducing, or anaerobic conditions [23]. Conversely, ferric iron uptake systems are common in marine organisms and comprise three different mechanisms: siderophore-mediated uptake, direct iron reduction, and uptake via transport proteins. Siderophores are small molecules with a high iron (III) binding affinity, and their biosynthetic genes are among the most prevalent in ocean microbial metagenomes, exhibiting high phylogenomic distribution [24]. The first marine siderophores, bisucaberin and anguibactin, were characterized in the late 1980s [25, 26]. Since then, multiple siderophores have been identified in seawater from metagenomic, metatranscriptomic, and metabolomic studies [27–31]. They collectively account for at least 0.2%–10% of the oceanic dissolved iron pool, evidencing the role that marine microbes play in iron cycling and speciation [27, 28]. Amphibactins and petrobactin are of particular interest, as they are some of the most abundant and widely distributed siderophores in the ocean [28, 29, 32]. Structurally, siderophores are classified by the specific substructures they use to chelate iron. The most common substructures are catecholate, hydroxamate, and α-hydroxycarboxylate (Fig. S1). Additional substructures include arylthiazoli(di)ne, aryloxazoli(di)ne, and diazeniumdiolate [33, 34]. However, siderophores often contain multiple of these substructures, thereby being classified as mixed siderophores [35].

The work presented in this manuscript is centered around discovery of metallophores involved in iron chelation in *Microbulbifer* sp. CNSA002 [36]. With this work, we expand upon the known *Microbulbifer* natural product repertoire by characterizing the first metallophore systems in this genus, including a new metallophore family we have named bulbichelins. We additionally describe unprecedented longer chain length acylation on the central spermidine moiety of petrobactin, which is one of the most abundant and widely distributed siderophore characterized from marine environments. These compounds were only produced in monoculture and not in mixed culture with the marine pathogen, *V. coralliilyticus*. We posit that deciphering how these metallophores shape *Microbulbifer*’s interspecies interactions and the regulation of their production will contribute to the understanding of their function in microbial chemical ecology.

## Materials and methods

Detailed methods are included in the supplementary information.

### Bacterial culturing for untargeted metabolomics experiments

Bacterial culturing was previously described [11]. Briefly, strains were cultured in SWB (saltwater broth) with or without ethylenediamine-*N,N′*-diacetic acid (EDDA). Harvested cultures were extracted using liquid–liquid extraction with ethyl acetate (EtOAc), solid phase extraction with C18 columns and solid liquid extraction with 2:2:1 ethyl acetate:methanol:water (EtOAc:MeOH:H_2_O). Extracts were dried *in vacuo*.

### Untargeted metabolomics data acquisition and analysis

Untargeted metabolomics data was acquired as previously described [11] and is detailed in the supplementary information. A mass spectrometry-based query language (MassQL) search was conducted in GNPS for the 30 siderophore substructures (Tables S1, S2). The results from each query were extracted and visualized using classical networking in GNPS. A MASST search was conducted in GNPS; datasets were reanalyzed, generating classical networks to identify related features.

### Fermentation, extraction, and purification of bisbulbichelin

*Microbulbifer* sp. CNSA002 was cultured in marine broth (MB/2) supplemented with EDDA. Harvested cultures were extracted with EtOAc. Extracts were dried *in vacuo* to yield a 0.5 g crude extract. The crude extract was fractionated using reversed phase liquid chromatography (RPLC). Fraction 4 contained bisbulbichelin (compound **1**) (1.0 mg).

Compound **1**: white powder. ^1^H and ^13^C NMR data, see Table S3. Positive HRESIMS *m/z* 1083.220 [M + H]^+^ (calculated for C_44_H_59_N_8_O_8_S_8_^+^, 1083.2217).

NMR data for compound **1** has been deposited to NP-MRD (ID: NP0352519).

### Fermentation, extraction, and purification of acyl petrobactins

*Microbulbifer* sp. CNSA002 was cultured in SWB and the cell-free supernatant and cell pellet were extracted. Both extracts were combined and the solvent was dried *in vacuo* to yield a 5 g crude extract. The crude extract was fractionated *via* RPLC to yield:

Compound **2**: yellow, oily. ^1^H and ^13^C NMR data, see Table S4. Positive HRESIMS *m/z* 873.497 [M + H]^+^ (calculated for C_44_H_69_N_6_O_12_^+^, 873.4968).

Petrobactin: yellow, oily. ^1^H and ^13^C NMR data, see Table S5. Positive HRESIMS *m/z* 719.361 [M + H]^+^ (calculated for C_34_H_51_N_6_O_11_^+^, 719.3610).

NMR data for compound **2** has been deposited to NP-MRD (ID: NP0351565).

### O-CAS agar assay

We performed a modified O-CAS [37] as previously described [11]. A colony of *Microbulbifer* sp. CNSA002, as well as the protein and small molecule fraction from spent media were tested. Compound **2** and fractions F14.15 and F14.16 were tested as well.

### Genome sequencing and analysis

AntiSMASH analysis of the *Microbulbifer* sp. CNSA002 genome (NCBI:txid3373604) predicted an NRPS metallophore and an NI-siderophore BGC.

We used the CAGECAT [38] clinker tool to compare the BGC from *Microbulbifer* sp. CNSA002 and known aryl thiazoli(di)ne/oxazoli(di)ne BGCs. Homologous proteins to BbchE were searched using BLAST. The 100 most similar sequences were used to generate a phylogenetic tree. Thioesterase (BbchB) phylogenetic analysis was generated, and family nomenclature was assigned based on the ThYme database.

The NIS BGC contained two *iucA/iucC* genes which were analyzed with EFI-EST and EFI-GNT [39] (enzyme function initiative-enzyme similarity tool and genome neighborhood tool). Protein co-occurrence data was binarized and mapped onto the phylogenetic tree.

We used the CAGECAT [38] clinker tool to compare the NI-siderophore BGC from *Microbulbifer* sp. CNSA002 and published petrobactin BGCs. Additionally, genes in the petrobactin cluster were queried against CNSA002 using BLAST.

### Direct metal infusion analyses

Relative binding assays were analyzed using a Thermo Scientific Q Exactive HF with upstream Vanquish UHPLC using a C18 UHPLC column. All metal solutions were prepared at concentrations of 2 and (where applicable) 0.5 mM. Raw data files were converted to the mzML format as previously described [40]. MS data was processed with mzmine 4.7.29 to produce feature tables. A binding percentage was calculated for each infused metal and concentration. All files relating to this analysis (e.g. mzML, mzmine batch, python code, excel) are deposited in MassIVE and in Github.

## Results and discussion

### Siderophore production by *Microbulbifer* sp. CNSA002

To investigate the ability of *Microbulbifer* sp. CNSA002 to obtain iron, we analyzed its genome for the presence of iron chelating proteins, and BGCs for metallophore biosynthesis. Genome analysis revealed the presence of multiple open reading frames encoding iron-binding and transport proteins (Table S6). This analysis showed that the CNSA002 strain has the genetic potential to import siderophores via TonB siderophore receptors, including receptor genes localized outside of BGCs. Additionally, two BGCs of interest were identified using the antiSMASH platform [41] (Fig. S2A). One BGC was predicted to encode a nonribosomal peptide (NRP), presenting low similarity (20%) to enantio-pyochelin. The other BGC was predicted to encode an NRPS-independent siderophore (NIS) [42]. Despite showing no similarity to known clusters, it was classified as such due to the presence of two *iucA/iucC* (iron uptake chelate) genes. To investigate the production of siderophores, we employed a modified chrome azurol S (O-CAS) assay [37] (Fig. S2B). The color change from blue to orange observed in and around the CNSA002 colony demonstrated its ability to produce iron chelators. To assess the presence of siderophores, we cultured *Microbulbifer* sp. CNSA002 in liquid media and fractionated the culture supernatant into the small molecule and macromolecule fraction. The color change in both the concentrated flow through and retentate indicated the production of iron-chelating compounds and smaller proteins (Fig. S2C).

### Mass spectrometry query language-based identification of features of interest

To identify the siderophores produced by CNSA002, we cultured this strain in complex media with and without the supplementation of ethylenediamine-*N,N′*-diacetic acid (EDDA). Metabolites were extracted using three methods to capture a diversity of compounds followed by mass spectrometry (MS) data acquisition. The MS^2^ spectra were used to generate a feature-based molecular network (FBMN) in the global natural products social molecular networking (GNPS) [43] platform (see methods). In this network, the structurally similar features are grouped into clusters of connected nodes by quantifying MS^2^ spectral similarity. This workflow additionally aids in the annotation of known compounds, as it computes the similarity between the input data and the GNPS MS^2^ spectral library, which consists of over 500 000 spectra. Despite our search against this MS^2^ spectral database and other compound databases such as MarinLit and dictionary of natural products, no matches to known siderophores or MS^2^ spectra were identified.

**Figure 1:**
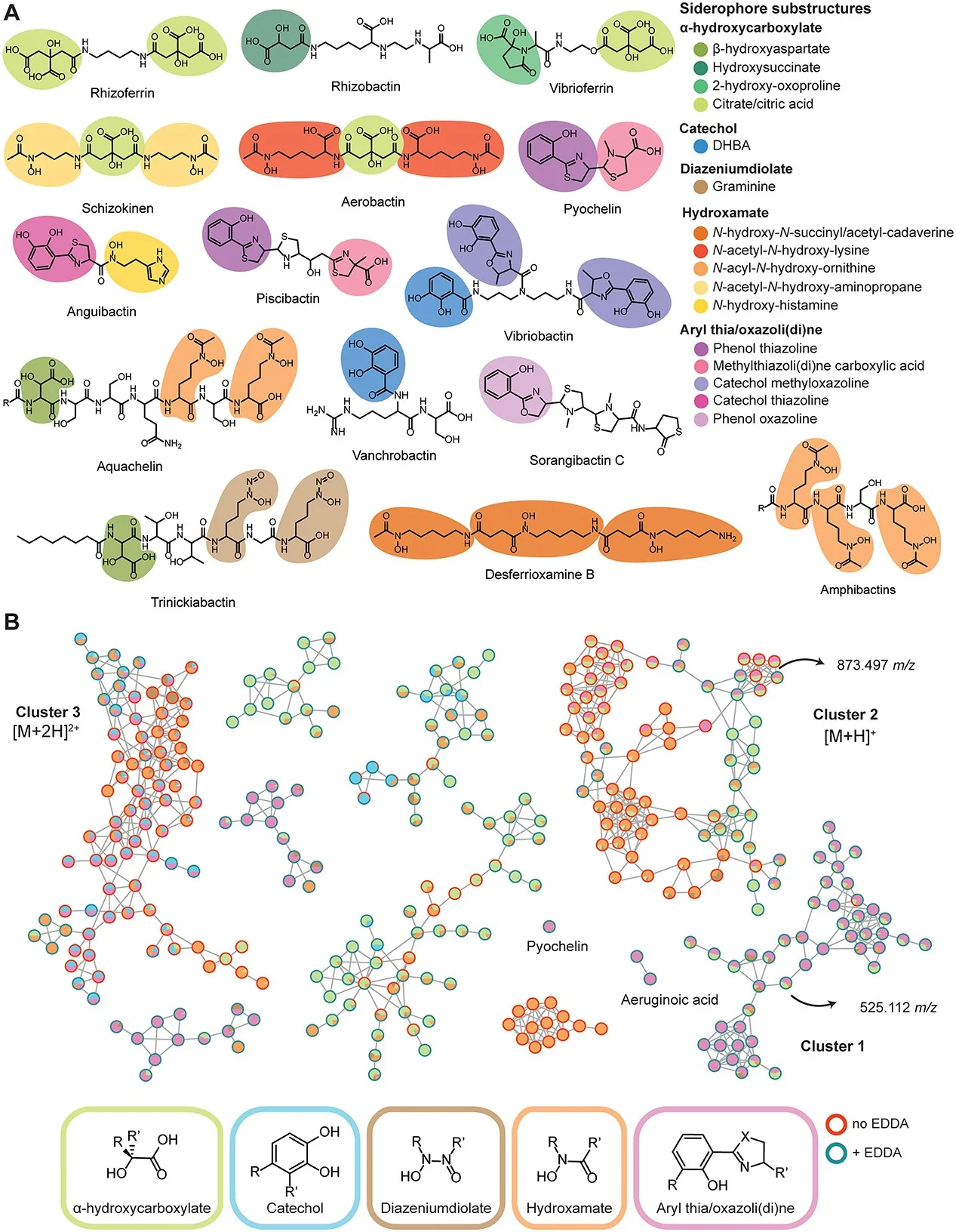
(A) Representative substructure library for MassQL searches. Siderophore families are grouped by color scheme and specific moieties are represented in different colors. DHBA: dihydroxybenzoic acid. (B) Representative FBMN of MassQL results. Spectral matches to non-siderophore compounds were subtracted, yielding a network of 1812 nodes. Clusters of interest and features at *m/z* 525.112 and 873.497 are labeled. Siderophore families are shown, R and R’ represent diverse aryl or alkyl groups, X = S or O. Node pie charts represent the proportion of query hits for each family and border color represent culturing conditions (with or without EDDA).

Therefore, we applied MassQL [44] to identify putative siderophores. MassQL uses defined patterns of parent *m/z* (MS^1^) and fragmentation spectra (MS^2^) such as intensity of fragment ions, isotopic patterns, and neutral losses, to find specific classes of compounds. The isotopic pattern of Fe, and the mass shift between iron-bound and iron-free ligands, makes siderophores amenable to MS identification [45–47]. These two characteristics, easily translatable into MassQL queries, underpin the proposed use case for siderophore search using the tool [40]. However, this query did not result in any matches within our dataset, presumably due to lack of detection of iron-bound siderophores. Therefore, we generated a comprehensive library of siderophore substructures (Fig. 1A, Table S1) and wrote queries to mine our dataset for presence of such substructures (Table S2). Siderophore discovery through MS^2^ mining has been previously implemented in MATLAB [48], however, the approach described herein covers a larger and comprehensive library of substructures and employs a web-browser-based tool [44]. The 30 substructures employed belong to five different families, namely, α-hydroxycarboxylate, catechol, diazeniumdiolate, hydroxamate, and aryl thiazoli(di)ne/oxazoli(di)ne (Fig. 1A, Table S1). The results from each substructure were analyzed using molecular networking in GNPS and visualized in Cytoscape [49]. In this network, we identified three clusters of interest (Fig. 1B), which were further characterized through BGC and MS^2^ analysis, described below. Notably, cluster 1 was uniquely detected under iron-limited conditions (EDDA addition), whereas clusters 2 and 3 were detected in rich media (without EDDA) to a greater relative abundance.

### Cluster 1: NRPS metallophore

The cluster 1 had matches to the phenol thiazoline, 2-hydroxy-5-oxoproline, *N*-formyl-*N*-hydroxy-ornithine and β-hydroxyasparagine substructure queries in MassQL output (Fig. S3A). The putative identification of a phenol thiazoline substructure allowed us to attribute these features to the NRPS metallophore BGC, while other substructure matches were identified as false positives and are discussed in the supplementary text. Literature searches and database searches for this siderophore family, the phenol thiazoline substructure and the corresponding neutral mass, did not yield matches. Additionally, these features had no MS^2^ matches to known compounds in the GNPS spectral library and MS^1^ matches to other databases such as SIDERITE [50]. We then used the MASST [51] tool to search for the MS^2^ spectra of this unknown feature against MS^2^ datasets in the MassIVE dataset repository. MASST is a web-based tool that allows users to search for identical or similar MS^2^ spectra in data repositories, regardless of whether the compound is annotated. This search didn’t result in analogous spectra either, which prompted us to characterize this compound as a potentially new metallophore.

**Figure 2:**
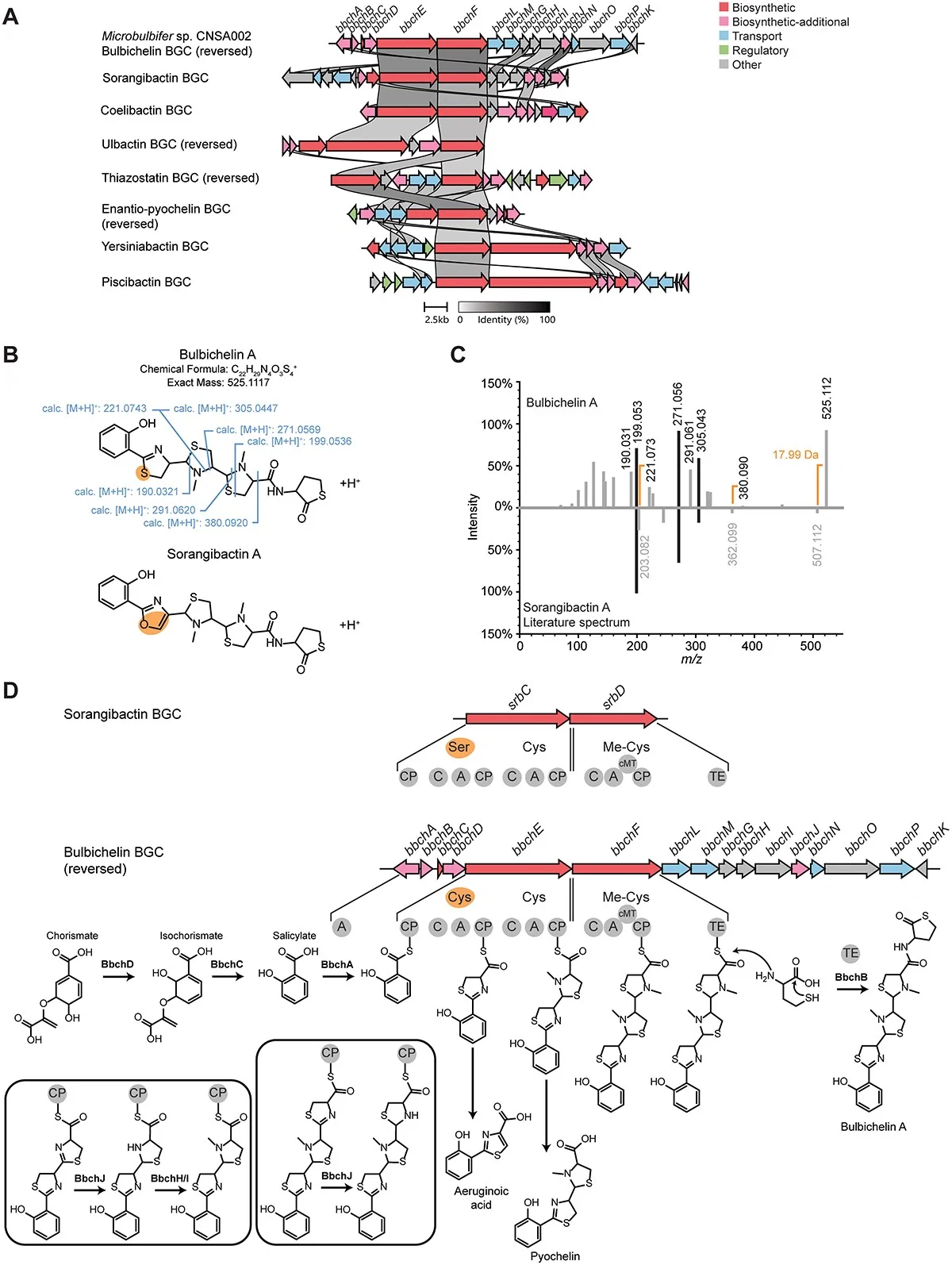
(A) Synteny analysis of aryl thia/oxazoli(di)ne siderophore BGCs. (B) Structure of bulbichelin A and sorangibactin A. Fragmentation is shown for bulbichelin A and structural differences are highlighted in orange. (C) Mirror plot comparing fragmentation spectra of bulbichelin A and sorangibactin A. (D) NRPS module architecture, adenylation domain prediction comparison for bulbichelin (*bbch*) and sorangibactin (*srb*) and proposed bulbichelin biosynthesis.

To inform the structural characterization of compounds in cluster 1, we performed a synteny analysis of the BGC from CNSA002 and aryl thia/oxazoli(di)ne metallophore BGCs (Figs 2A and S4) using the CAGECAT [38] clinker tool, with the results showing good alignment of the core biosynthetic genes. AntiSMASH [41] had predicted the most similar known cluster to this BGC to be enantio-pyochelin, however, the synteny and phylogenetic analyses revealed a higher similarity to the sorangibactin BGC [52] (Figs 2A and S5). Given its high relative abundance (area under the chromatographic peak), we selected the feature at 525.112 *m/z* (predicted molecular formula, C_22_H_29_N_4_O_3_S_4_^+^) as a representative feature for further investigation. Based on BGC and MS^2^ spectral similarity between sorangibactin A and the 525.112 *m/z* feature produced by CNSA002, we proposed its structure and named it bulbichelin A (Fig. 2B). Spectral analysis supports the similarity between bulbichelin A and sorangibactin A (Fig. 2C). Notably, two fragment peaks (221.073 and 380.093 *m/z*) and the parent ion exhibit a mass shift of 17.99 Da when compared to the sorangibactin A spectrum. Additionally, substrate prediction in antiSMASH for the adenylation domains in these two BGCs (sorangibactin BGC, *srb*, and bulbichelin BGC, *bbch*) differ in the incorporation of a cysteine in bulbichelin in place of serine in sorangibactin (incorporated by BbchE and SrbC, respectively) (Fig. 2D). This biosynthetic discrepancy supports the structural and spectral differences observed. Indeed, this Δ*m/z* corresponds to the incorporation of O in place of S (−15.9771 Da) and loss of one degree of unsaturation (−2.014 Da). The discovery of sorangibactins brought forward the unprecedented incorporation of a thiolactone at the C-terminus [52], which is also present in bulbichelin with an additional substitution of oxazole in sorangibactin A to thiazoline in bulbichelin A.

To analyze the distribution of similar biosynthetic enzymes, a phylogenetic tree was constructed incorporating the 100 most similar sequences to BbchE (Fig. S5). The tree suggests a close relationship between the *Sorangium* sp. and *Microbulbifer* sp. clades, underscoring the similarity of their biosynthetic products. The phylogenetic tree also revealed anthrochelin (*ant*) as an additional BGC of interest, yielding biosynthetic products that are structurally related to sorangibactins [53]. Furthermore, the tree highlights the prevalence of BbchE in the *Microbulbifer* genus, being identified in 34 publicly available genomes. This is consistent with a recent phylogenetic inventory, which identified this gene cluster to be present in 38 *Microbulbifer* sp. genomes [6].

Sequence homology and gene analysis was used in this study to propose a biosynthesis scheme for bulbichelin, which contains an N-terminal phenol, a thiazoline, two *N*-methyl-thiazolidines, and the C-terminal γ-thiolactone moiety (Fig. 2D). This BGC is composed of 16 annotated proteins involved in biosynthesis and transport (Table S7). Biosynthesis is initiated by salicylate formation from chorismate, proposed to be catalyzed by BbchD and BbchC. These enzymes are predicted to be an isochorismate synthase and lyase, respectively. The salicylate moiety is then adenylated by BbchA and loaded onto the first BbchE carrier protein. BbchE and BbchF comprise three adenylation and heterocyclization domains, predicted to use cysteine as substrates for chain elongation. In addition to these core enzymes, BbchH-BbchJ are tailoring enzymes, working “in *trans*” as oxidoreductases and methyltransferases (Table S7). Chain release is then carried out by a thioesterase (TE) domain. This TE likely uses homocysteine as an intermolecular nucleophile, as has been observed for select TEs [52–54]. The intermolecular offloading reaction yields a linear product, and we hypothesize that a stand-alone TE (BbchB) carries out the thiolactonization of the homocysteine to yield bulbichelin A (Fig. 2D). Notably, the known metallophores pyochelin and aeruginoic acid were also detected upon iron limitation (Fig. 1B). Given the similarity of these compounds to bulbichelin, we hypothesize that these are shunt products of the bulbichelin BGC, which is consistent with the proposed biosynthesis (Fig. 2D).

This proposed biosynthetic route was compared to sorangibactin and anthrochelin biosynthesis. In anthrochelin, salicylate formation is carried out by AntN and AntM respectively [53], homologous enzymes to BbchD and BbchC. This process differs from the proposed biosynthesis for sorangibactin, where salicylate is synthesized from chorismate via a one-step reaction, catalyzed by the salicylate synthase SrbB [52]. Regarding offloading, similar reactions utilizing homocysteine as a nucleophile are proposed for both sorangibactin and anthrochelin, carried out by SrbD and AntB. Multiple sequence alignment comparing the TE domains in SrbD, AntB and BbchF revealed a conserved use of the rare catalytic cysteine, instead of serine, as the nucleophile in the enzyme active site (Fig. S6). The final thiolactonization step has remained unclear for both sorangibactins and anthrochelins [52, 53]. A homologue of BbchB, our proposed cyclizing TE, is present in the *ant* BGC (AntL), however, no homologues were identified in the *srb* BGC. Stand-alone thioesterases (type II TEs) are usually not involved in biosynthesis, performing an editing role instead by removing unsuitable substrates that block the assembly line [55]. Still, some type II TEs perform non-canonical functions [56]. For instance, SncF catalyzes a lactam-forming cyclization to yield the product spinactin A [57]. To support our proposal, we carried out a sequence analysis, including TEs with known crystal structure in the ThYme database [58], in addition to SncF, BbchB, and AntL. The ThYme database is a continuously growing compilation of thioesterases, grouping these enzymes into 35 families based on sequence similarity [58]. The output from this analysis shows clustering of these three TEs within the TE 18 family (Fig. S7), which supports our proposal of thiolactonization being carried out by BbchB; however, *in vitro* experiments are required to validate this hypothesis.

**Figure 3:**
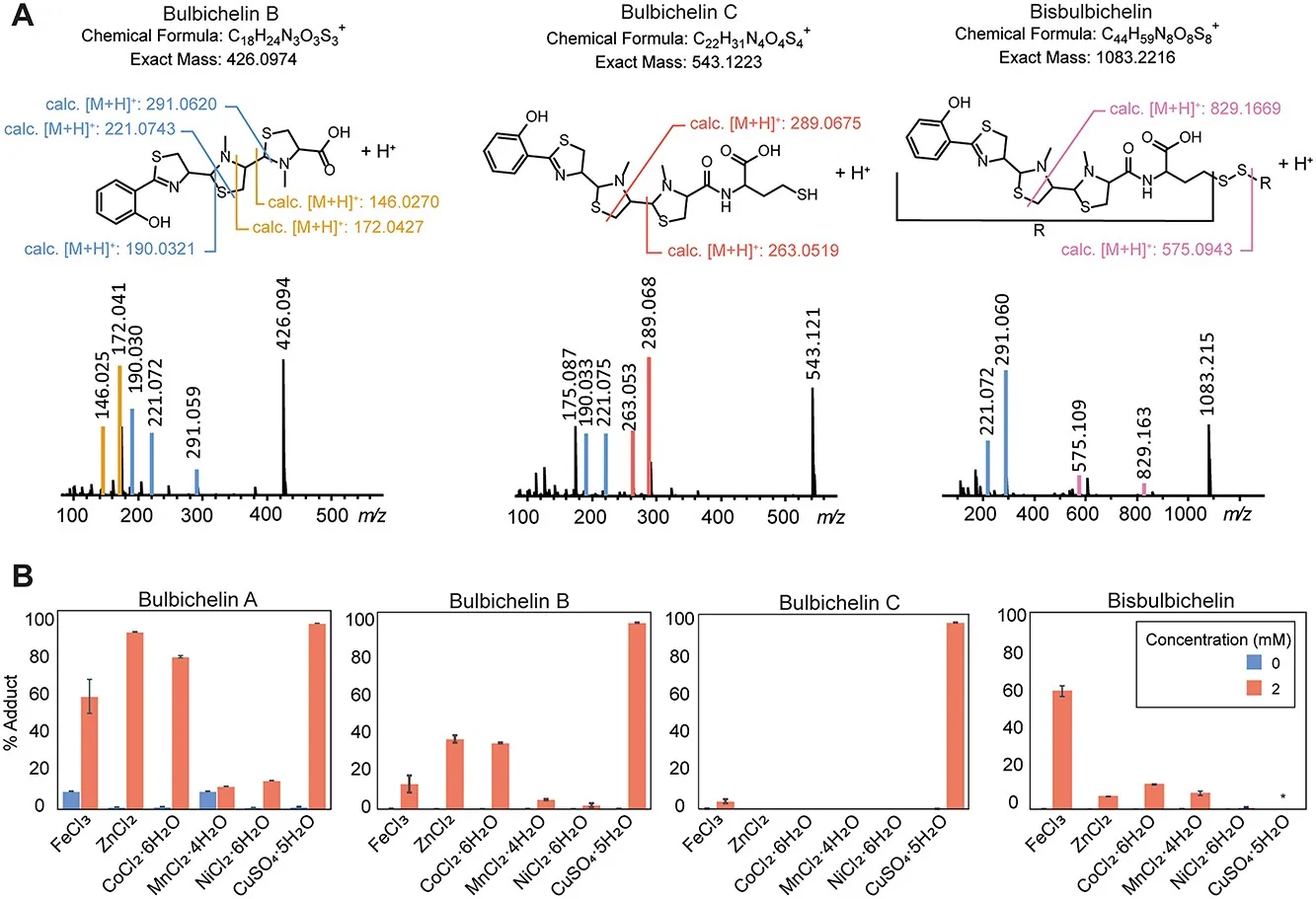
(A) Bulbichelin analogs MS^2^ fragmentation. NMR data for bisbulbichelin is included as supplemental information. (B) Bulbichelin analogs metal binding in direct infusion experiments. Binding percentage (% adduct) is defined as the peak area of the metal adduct divided by the total peak area of the metal adduct and the [M + H]^+^ adduct in each infusion run. N = 3 for each water infusion (control 0 mM; blue bars) and metal infusion (2 mM; red bars). Several copper adducts are indicated as 100% because proton-bound peaks were either not found or were below the limit of detection, suggesting complete conversion. The asterisk indicates that bisbulbichelin exhibits the adduct bound to two copper ions along with potential reactivity with copper.

The similarity between the bulbichelin, anthrochelin, and sorangibactin BGCs*,* revealed through synteny and phylogenetic analyses, informed the dereplication of bulbichelin analogs (Figs 3A and S8, Table 1). Bulbichelin B bears resemblance to sorangibactin D and anthrochelin B, harboring a carboxylic acid on the C-terminal end and suggesting a hydrolytic release from BbchF TE domain. Bulbichelin C, on the other hand, bears similarity to sorangibactin B and anthrochelin C, where the linear homocysteine suggests it is an intermediate product, prior to the formation of thiolactone. This linear homocysteine could also stem from hydrolytic ring opening due to chromatographic conditions. Interestingly, this thiol group can form disulfide bonds through oxidation, as observed in bisanthrochelin. Bulbichelin C is also subject to this reaction resulting in dimeric bisbulbichelin. Given the proclivity to both ring opening and dimerization during the isolation process, bisbulbichelin was the most amenable to structural confirmation via nuclear magnetic resonance (NMR) (Figs S9–S13, Table  S3).

**Table 1:** Bulbichelin analogs detected in *Microbulbifer* sp. CNSA002 iron-limited culture.

Name	Adduct	Theoretical *m/z*	Experimental *m/z*	Error (ppm)
Bulbichelin A	[M + H]^+^	525.112	525.112	0
Bulbichelin A	[M + 2H]^2+^	263.060	263.059	4
Bulbichelin B	[M + H]^+^	426.097	426.098	2
Bulbichelin C	[M + H]^+^	543.122	543.123	2
Bulbichelin C	[M + 2H]^2+^	272.065	272.064	4
Bisbulbichelin	[M + H]^+^	1083.222	1083.220	2
Bisbulbichelin	[M + 2H]^2+^	542.115	542.113	4

#### Metal binding by bulbichelins

The metal binding properties of bulbichelins were determined using MS. Direct metal infusions allowed for the detection of iron-bound bulbichelins, supporting their potential role as siderophores (Figs 3B and S14). These iron complexes exhibit the characteristic Δ*m/z* of 52.911 Da, stemming from the [M + Fe-2H]^+^ adduct, and distinct isotopic pattern reflecting the stable isotope ratio between ^54^Fe and ^56^Fe (Fig. S14). Bulbichelin A exhibited the highest iron-binding, while analogs B, C, and bisbulbichelin exhibited lower iron-binding percentages (Fig. 3B). Here, the binding percentage is defined as the metal-adduct peak area divided by the total peak area of the metal adduct plus the [M + H]^+^ adduct in each infusion run. Notably, the chelates display a red-orange color due to strong ligand-to-metal charge transfer bands in the UV–Vis region (HPLC vial insert in Fig. S14A). Additional direct metal infusion experiments were carried out with a panel of transition metals, given the demonstrated metallophore capabilities of the aryl thiazoli(di)ne/oxazoli(di)ne family [59–61]. In these native infusion MS experiments, bulbichelins also demonstrate metal-binding promiscuity (Fig. 3B). Bulbichelins A, B, and C exhibit appreciable copper binding, and bisbulbichelin shows binding to two copper ions (Fig. S15). Furthermore, bulbichelin A exhibited high binding percentages for zinc and cobalt. Bulbichelin B also binds these two metals, though perhaps less readily based on lower binding percentages (Fig. 3B), while bulbichelin C and bisbulbichelin show low to negligible binding of these two metals. Interestingly, bulbichelins A and B show higher binding ratios of zinc, cobalt, and copper as compared to iron. Finally, while bulbichelin A and bisbulbichelin demonstrate low nickel and manganese binding, any nickel or manganese binding to bulbichelins B and C is below the limit of detection for these experiments, suggesting minimal binding (Fig. 3B). Thus, bulbichelins may serve as broad spectrum metal binders, a beneficial trait in the dilute marine environment.

### Clusters 2 and 3: NI-siderophore

Clusters 2 and 3 represent [M + H]^+^ and [M + 2H]^2+^ adducts of the same compounds and were therefore analyzed in parallel. These clusters had MassQL hits belonging to dihydroxybenzoic acid, graminine, catechol methyloxazoline, citrate, and several hydroxamate queries (Fig. S3B). The presence of catechol in these features was additionally evidenced by the characteristic 3,4-dihydroxybenzoic acid UV absorbance ratio at 294 nm and 255 nm^62^ (Fig. S16). The putative identification of citrate substructures allowed us to connect these features to the NI-siderophore BGC predicted by antiSMASH, given that citric acid is a common substrate in NI-siderophore biosynthesis [42]. As pointed out for bulbichelins, some of the MassQL query hits were identified as false positives and are discussed in the supplemental material. No nodes in these clusters had matches to known siderophores in compound databases nor the GNPS spectral library. Therefore, we performed a MASST search to look for similar MS^2^ spectra in publicly available MS^2^ datasets, regardless of prior identification. One dataset (Fig. S17) contained a MassQL query hit to the known NI-siderophore petrobactin. Therefore, we hypothesized that these features are structurally related to petrobactin and annotated them through MS and NMR analysis. This hypothesis was additionally supported by genomic analysis of the NI-siderophore BGC, as described below.

#### Annotation of acyl petrobactins

**Figure 4:**
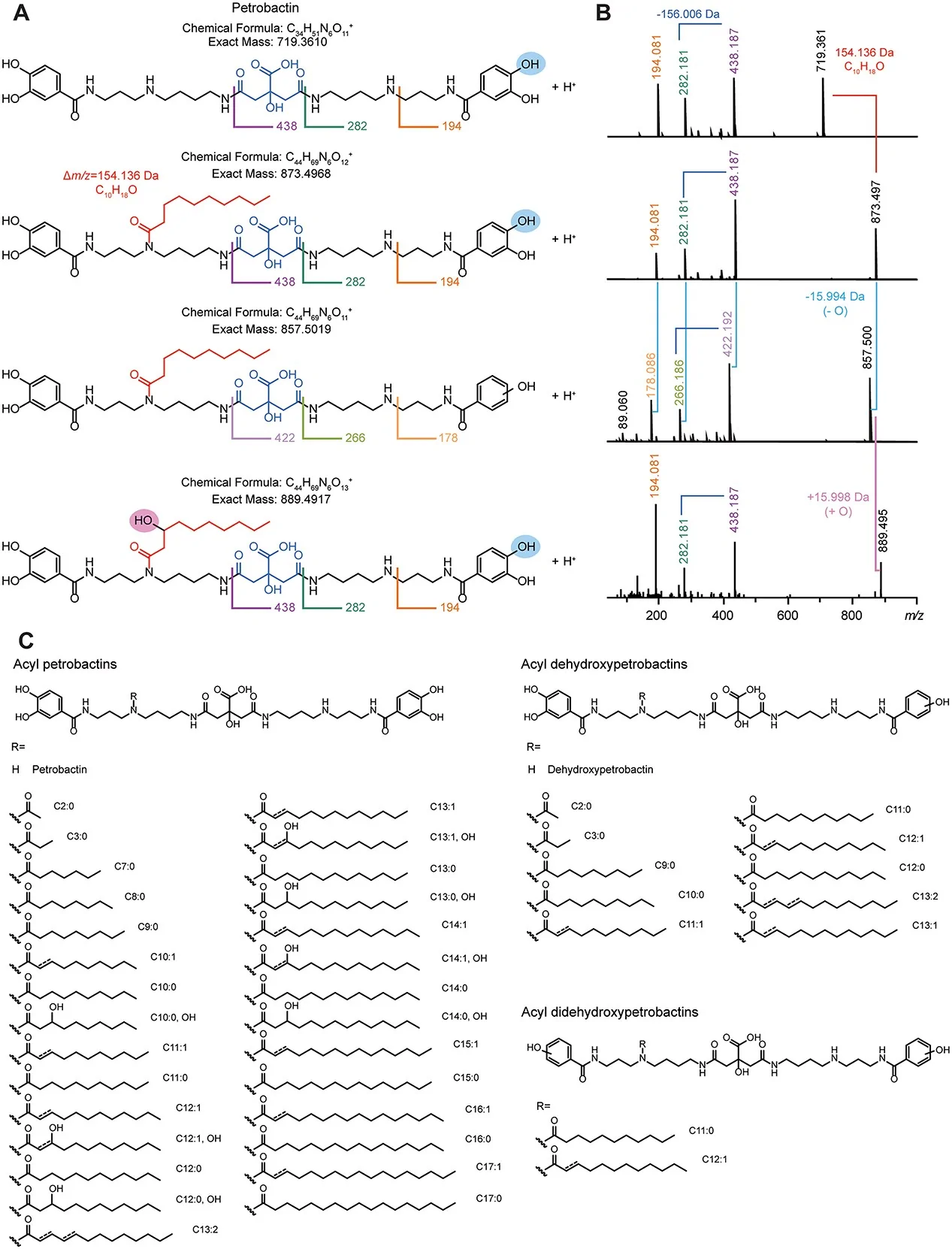
(A) Representative structure of acyl petrobactins, acyl dehydroxypetrobactins and hydroxyacyl petrobactins and comparison with petrobactin headgroup. Acyl tails are shown in red with the corresponding mass shift. MS^2^ fragments and hydroxyl functional groups are highlighted. (B) MS^2^ comparison between acyl petrobactins, acyl dehydroxypetrobactins and hydroxyacyl petrobactins, mass shifts are shown in light blue and pink. (C) Suites of acyl petrobactins, acyl dehydroxypetrobactins, and acyl didehydroxypetrobactins.

To annotate these petrobactin analogs, we compared the MS^2^ of our features of interest with the petrobactin MS^2^. Given its high relative intensity, we selected the feature at 873.497 *m/z* (predicted molecular formula, C_44_H_69_N_6_O_12_^+^) as a representative feature for this cluster. The mass shift between this feature and petrobactin corresponds to C_10_H_18_O, congruent with a C10:0 acylation of petrobactin (Fig. 4A and B). Similar mass shifts, indicative of acylations, were annotated for other features in the cluster (Figs S18 and S19). Isolation of this compound and comparative analysis of NMR data between acylated and nonacylated petrobactin revealed an additional carbonyl carbon, confirming the presence of a new amide bond and its position within one of the spermidine moieties (Tables S4 and S5, Figs  S20–S25).

Following the structural elucidation of petrobactin C10:0, we propagated the annotations for additional analogs in the molecular network through MS^2^ analysis (Fig. 4C, Table S8). Herein, we identified a suite of petrobactins with varying acyl tail lengths (C2,3,7–17), as well as modifications in the acyl tail such as hydroxylation and unsaturation. Acylated siderophores have been reported from both marine and terrestrial sources [62–65], however, acylations are particularly prevalent in aquatic environments, where diffusion of these secondary metabolites represents a high metabolic cost [66]. Acyl tails can act as a tether to the cell membrane [63], limiting diffusion and facilitating receptor binding [67]. Additionally, diversity in acylating groups creates variability in siderophore hydrophobicity, and the different analogs partition based on acyl tail length, saturation and functionality [68–70]. Acylated petrobactins exhibit this membrane partitioning, as longer acyl tail analogs are exclusively detected in the cell extracts, whereas short-chain analogs are only detected in the supernatant extracts (Table S8). Although acylated siderophores are common, the position of the acylation on the central secondary amine observed for petrobactins is rare, as most siderophore acylations are terminal (Fig. S26). The only exception is observed in the siderophore family rhodopetrobactins [71] and the metallophore methylolanthanin [72]. However, these compounds incorporate acetylations, thus, the longer chain acylations (up to C17) described in this work on the central secondary amines are unprecedented in siderophores.

Additional analogs bearing modifications on the petrobactin headgroup were annotated, including the acyl (di)dehydroxypetrobactins, which exchange one or both catechols for a phenol (Fig. 4A and C). The absence of one hydroxylation renders the dehydroxypetrobactins an asymmetrical molecule, allowing for the detection of both catechol and phenol fragments in MS^2^ (Fig. S19). On the other hand, didehydroxypetrobactins are symmetrical and only phenol fragments are observed. The incorporation of a phenol instead of a catechol is uncommon for this type of siderophores, as this moiety only contributes one denticity to the molecule, therefore losing the preferred hexadenticity for iron chelation. Siderophores with phenolate moieties usually include them as a part of aryl thiazoli(di)ne/oxazoli(di)ne substructures as discussed above for bulbichelin. Since the BGC includes a 3-dehydroshikimate dehydratase, which converts this substrate into 3,4-dihydroxybenzoic acid [73, 74], it is unlikely for these analogs to be intermediates in acyl petrobactin biosynthesis. Instead, these phenol moieties could stem from the ubiquinone pathway, where chorismate is converted to 4-hydroxybenzoic acid by the chorismate-pyruvate lyase UbiC [75].

**Figure 5:**
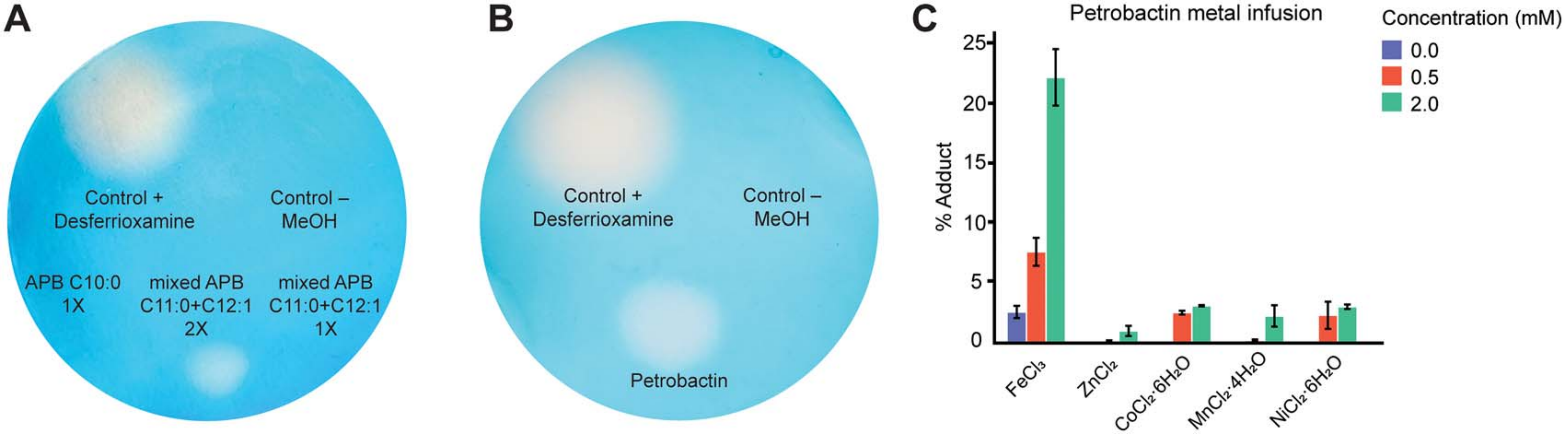
(A) O-CAS assay for acyl petrobactin (APB) fractions. (B) O-CAS assay for isolated petrobactin. (C) Metal binding by petrobactin in direct infusion experiments. Binding percentage (% adduct) is defined as the peak area of the metal adduct divided by the total peak area of the metal adduct and the [M + H]^+^ adduct in each infusion run. N = 3 for each metal infusion and metal concentration (0, 2, and 5 mM).

Despite uncommon, these structural changes have been observed in rhodopetrobactins and the structurally related methylolanthanin. Methylolanthanin incorporates two phenols and is therefore a tetradentate ligand. Despite this denticity, it is still able to chelate iron, albeit with low affinity [76] and the compound is primarily described as a lanthanophore [72]. These findings raise the question of whether petrobactin and its analogs can chelate other metals. We confirmed the ability of (acyl)petrobactin to chelate iron through the same O-CAS agar assay (Fig. 5A and B) and tested their affinity for additional transition metals (Zn, Co, Mn, and Ni) through direct metal infusion, however, there was a clear preference for Fe (Figs 5C and S27).

**Figure 6:**
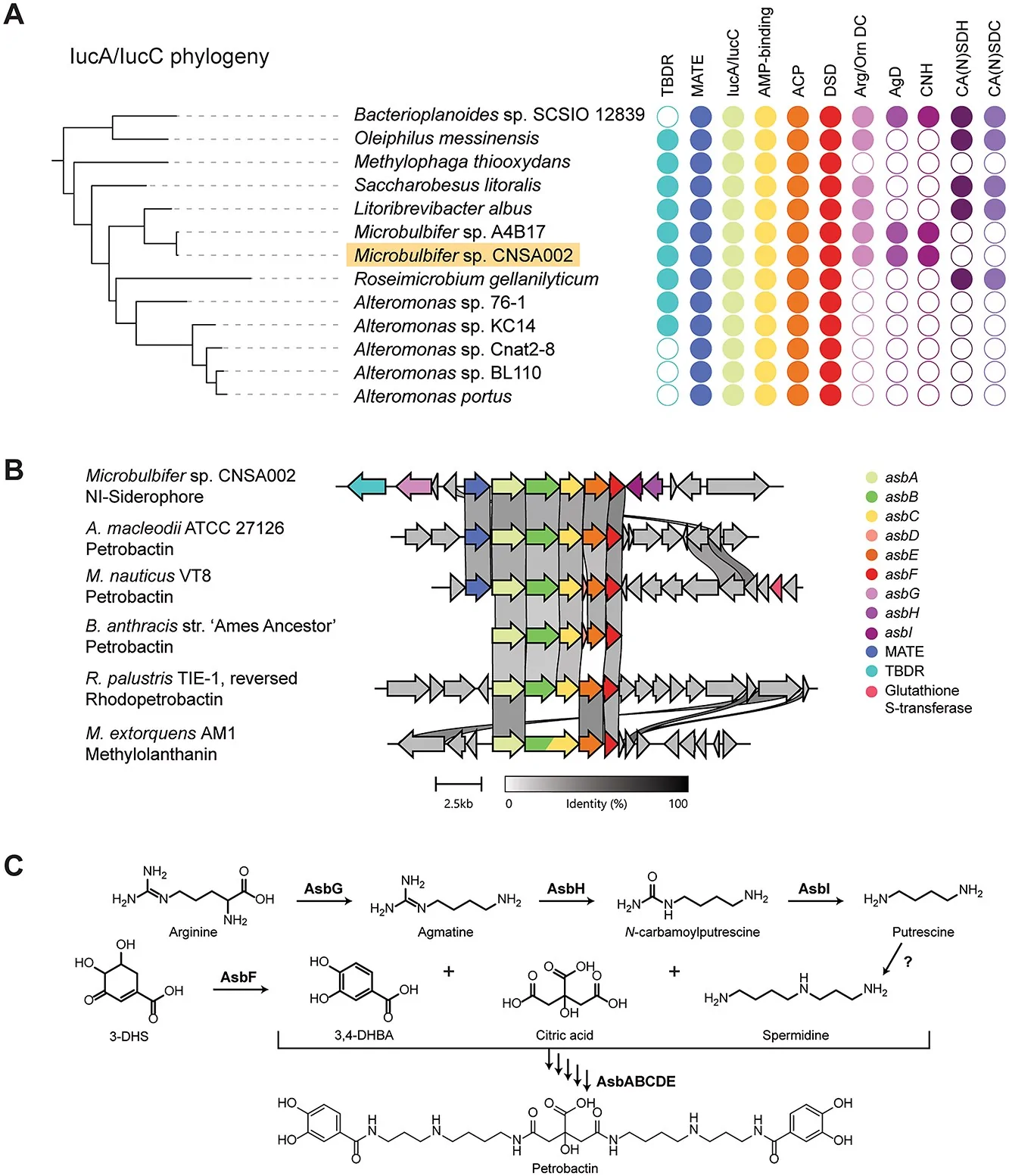
(A) IucA/IucC protein phylogeny and co-association analysis. Circles denote presence/absence in gene neighborhood. TBDR: TonB-dependent receptor; MATE: MATE (multidrug and toxic compound extrusion) family efflux transporter; IucA/IucC: IucA/IucC siderophore biosynthesis; ACP: Aryl-carrier protein; DSD: 3-dehydroshikimate dehydratase; Arg/Orn DC: arginine/ornithine decarboxylase; AgD: agmatine deiminase; CNH: carbon-nitrogen hydrolase; CA(N)SDH: carboxy(nor)spermidine dehydrogenase; CA(N)SDC: carboxy(nor)spermidine decarboxylase. (B) Synteny analysis comparing the NI-siderophore BGC from *Microbulbifer* sp. CNSA002, siderophore clusters from petrobactin-producing bacteria and the published petrobactin BGC from *B. anthracis*. BGCs from the structurally related compounds rhodopetrobactin and methylolanthanin are also shown. (C) Petrobactin biosynthesis including AsbG: arginine decarboxylase, AsbH: agmatine deiminase, AsbI: *N*-carbamoylputrescine amidase. 3-DHS: 3-dehydroshikimate; 3,4-DHBA: 3,4-dihydroxybenzoic acid.

Petrobactin is a photoreactive siderophore, where the citrate moiety undergoes an oxidative decarboxylation [77]. However, we did not detect any acylated petrobactin photoproducts, despite no precautions being taken to prevent photodegradation. The sulfonated petrobactin analogs were also not detected in our work, which is unsurprising as our strain does not contain the glutathione *S*-transferase present in other petrobactin BGCs (Fig. 6B) [78, 79].

### Genomic analysis of *Microbulbifer sp.* CNSA002 NI-siderophore biosynthetic gene cluster

Genomic analysis of the NI-siderophore BGC, linked to clusters 2 and 3 in the molecular network, supported our initial identification of these features as petrobactin analogs. A phylogeny and co-association analysis for the biosynthetic enzymes (IucA/IucC) in this BGC was carried out. In this analysis, we identified the clade in which *Microbulbifer* sp. CNSA002 is nested (Fig. 6A) and used antiSMASH [41] to predict the NI-siderophores produced by the other organisms in the clade. Here, two BGCs presented similarity to the roseobactin BGC, although with low confidence (Table S9). Roseobactin is a citrate-catechol mixed siderophore produced by *Phaeobacter inhibens* that differs from petrobactin in the orientation of the spermidine moiety, ultimately exchanging the positions of the putrescine and 1,3-diaminopropane subunits [80] (Fig. S28). On the other hand, functional annotation of the CNSA002 genome using the RAST (Rapid Annotation using Subsystem Technology) platform [81] and SEED viewer [82] (Table S6) predicted the presence of anthrachelin biosynthesis genes (*asb* BGC). These genes were initially identified in *Bacillus anthracis* [83] but later studies determined the product of this BGC to be petrobactin [84], supporting our initial annotation for these features.

The petrobactin BGC (*asb*) is a hybrid NIS-NRPS BGC, bearing two enzymes from the NIS family (AsbA and AsbB), three belonging to the NRPS family (AsbC, AsbD, and AsbE) and a dehydroshikimate dehydratase (DSD, AsbF) [73, 74] (Table S10). Homologs of these enzymes co-occur in all the genomes in the CNSA002 clade (Fig. 6A). Other co-occurring genes include the transport-related TonB-dependent receptor and multidrug and toxic compound extrusion (MATE) family efflux transporter. Interestingly, the genomic neighborhoods in the co-association analysis also included polyamine biosynthetic genes. Comparison of the *asb* BGC from *Microbulbifer* sp. CNSA002 and other known petrobactin producers shows good alignment of the BGCs compared and additionally reveals the unique inclusion of polyamine biosynthesis genes in our strain (Fig. 6B). The *Microbulbifer* sp. CNSA002 *asb* includes an arginine decarboxylase (AsbG), an agmatine deiminase (AsbH) and an *N*-carbamoylputrescine amidase (AsbI), which sequentially produce putrescine (Fig. 6C).Previous BGC analyses have described petrobactin biosynthesis with spermidine as a substrate [85–87]. We additionally propose the steps involved in the biosynthesis of spermidine based on presence of biosynthetic enzymes in the *Microbulbifer* sp. CNSA002 BGC (Fig. 6B and C). From the co-association analysis, only one bacterium’s BGC in the clade (*Bacterioplanoides* sp. SCSIO 12839) incorporates all the necessary enzymes for spermidine biosynthesis from arginine. The genome of *Microbulbifer* sp. CNSA002 does encode a putative carboxyspermidine dehydrogenase and a carboxyspermidine decarboxylase elsewhere, responsible for spermidine biosynthesis from putrescine.

### Dysregulation of siderophore production upon coculture with *Vibrio coralliilyticus* Cn52-H1

**Figure 7:**
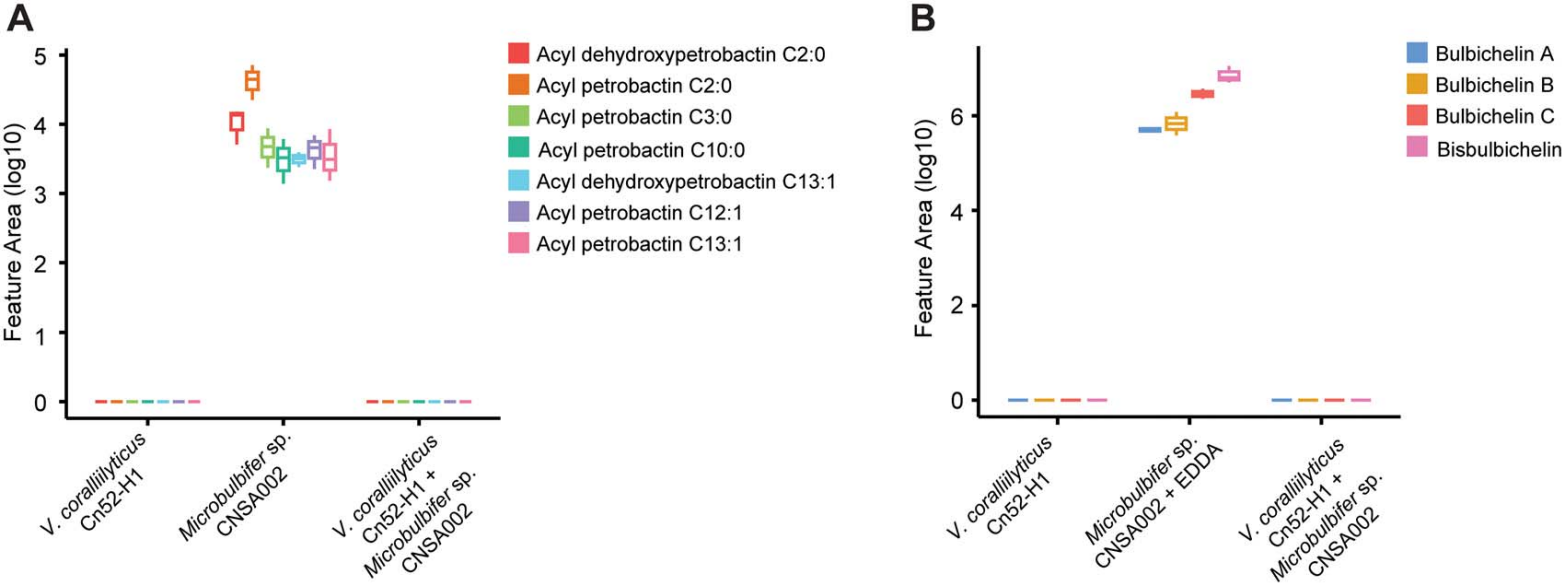
(A) Boxplots of the relative abundances of acylated petrobactins in *Microbulbifer* sp. CNSA002, the pathogen *Vibrio coralliilyticus* Cn52-H1 and their coculture. (B) Boxplots of the relative abundances of bulbichelins in *Microbulbifer* sp. CNSA002 grown in iron limited media (EDDA addition), the pathogen *Vibrio coralliilyticus* Cn52-H1 and their coculture. Relative abundances were obtained from LC–MS data.

We previously reported that *Microbulbifer* sp. CNSA002 enzymatically degrades the siderophore amphibactin produced by the marine pathogen *V. coralliilyticus*, and that the responsible enzyme is constitutively expressed, highlighting its ecological relevance [11]. These findings prompted us to investigate the iron acquisition strategies employed by *Microbulbifer*, and their implication in ecological interactions with competing marine bacteria. In this study, the (acyl)petrobactins and bulbichelins were only detected in the *Microbulbifer* sp. CNSA002 monoculture (Fig. 7A and B). At first glance, the apparent repression of metallophore production in the presence of a competing bacterium is counterintuitive, as iron limitation and interspecies competition typically stimulate siderophore biosynthesis [88]. One possible explanation is that *Microbulbifer* shifts from de novo siderophore production to siderophore piracy, a strategy described in several other marine bacteria [89]. Consistent with this hypothesis, genome analysis identified multiple TonB-dependent siderophore receptors and outer membrane receptors predicted to recognize ferric complexes of heterologous siderophores, including coprogen and rhodotorulic acid (Table S6), despite the absence of associated BGCs. Together with the previously described amphibactin-degrading enzyme, these receptors suggest that *Microbulbifer* may acquire iron by exploiting siderophores produced by neighboring microorganisms.

This hypothesis is further supported by the outcome of a growth assay performed in iron-deficient seawater-based medium [90]. Here, *Microbulbifer* sp. CNSA002 failed to grow when cultured alone but exhibited robust growth in the vicinity of *V. coralliilyticus* Cn52-H1 (Fig. S29A). The ability of *Vibrio* to produce siderophores under these conditions was confirmed using the O-CAS overlay assay (Fig. S29B), suggesting that *Microbulbifer* can benefit from siderophores produced by its competitor. Similar siderophore-mediated growth rescue has been reported across diverse microbial systems under both iron-limited and iron-replete conditions [91–93].

Conversely, amphibactin degradation and the purported decrease in iron affinity and stability of the product [11] could promote release of bound iron from the chelate increasing iron concentration and repressing siderophore biosynthesis in CNSA002. If the amphibactin-degrading enzyme exhibits broad substrate specificity toward structurally related NRPS-derived siderophores, *Microbulbifer* could simultaneously access iron from multiple exogenous metallophores while circumventing iron sequestration by competing bacteria. Alternative explanations should also be considered. The absence of detectable acyl petrobactins and bulbichelins in coculture may reflect their conversion into previously unannotated metabolites, analogous to the disappearance of amphibactins following enzymatic degradation [11]. Failure to detect these compounds could also be due to their production under the instrumental limit of detection or unanticipated chelate stoichiometries and compositions, particularly for the metallophore bulbichelins. Although comparative metabolomics and substructure mining did not provide evidence for such products, limited metabolite abundance, altered metal-chelate stoichiometries, or changes in ionization efficiency may also have prevented their detection.

Collectively, these findings suggest that *Microbulbifer* possesses a flexible iron acquisition strategy that combines siderophore degradation, uptake of heterologous siderophores, and endogenous metallophore biosynthesis. Given the ecological importance of *Microbulbifer* in degrading complex marine polysaccharides, these adaptations may also position this genus as an important contributor to iron cycling within coastal microbial communities. Elucidating the regulation and environmental deployment of these complementary iron acquisition mechanisms will provide valuable insight into microbial competition and nutrient exchange in marine ecosystems.

## Supplementary Material

Supporting_information_ycag238

## Data Availability

NMR data for compound **1** has been deposited to NP-MRD (ID: NP0352519). NMR data for compound **2** has been deposited to NP-MRD (ID: NP0351565). LCMS data has been deposited in GNPS-MassIVE under dataset IDs MSV000096127, MSV000098865, MSV000099319, MSV000100161 and MSV000100952. BGCs have been deposited in MIBiG under accession numbers BGC0003186 (petrobactin) and BGC0003187 (bulbichelin). Spreadsheets, mzmine batch files, and other data related to metal infusions and analysis can be found at https://github.com/redghoti43/CNSA002_Metallophores_Paper_Data.

## Supporting information

Siderophore structure examples, MS^2^ and NMR analysis of acylated petrobactins, siderophore moieties used for MassQL queries, and *Microbulbifer* sp. CNSA002 genomic analysis.
