# Supplementary material for: Computational mass spectrometry and genome mining guided discovery of metallophores produced by Microbulbifer
Source: ISME Commun. 2026 Aug 19;6(1):ycag238. doi: 10.1093/ismeco/ycag238 (PMC13573596; doi:10.1093/ismeco/ycag238)
Supplement: Supplementary file 1 [file supporting_information_ycag238.docx]

**Computational mass spectrometry and genome mining guided discovery of metallophores produced by *Microbulbifer***

Mónica Monge-Loría^1^, Colin Brady^2^, Hongwei Wu^1^, Allegra Aron^2^, Neha Garg^1,3 *^

^1^School of Chemistry and Biochemistry, Georgia Institute of Technology. 950 Atlantic Drive, Atlanta, GA 30332 (USA).

^2^ Department of Chemistry and Biochemistry, University of Denver, Denver, CO (USA)

^3^Center for Microbial Dynamics and Infection, Georgia Institute of Technology. 315 Ferst Drive, Atlanta, GA 30332 (USA).

Correspondence: [neha.garg@chemistry.gatech.edu](mailto:neha.garg@chemistry.gatech.edu)

**Table of contents**

Discussion entailing false positives in the output of MassQL …..…….…….………………………………………………..3

Supplemental Methods…..………………………………………………………………………………………………………..3

[**Figure S1.** Examples of α-hydroxycarboxylate, catechol and hydroxamate siderophores. Iron-chelating moieties are circled. Mixed siderophores, incorporating more than one moiety, are presented as well.	8](#_Toc237877555)

[**Figure S2.** Production of siderophores by *Microbulbifer* sp. CNSA002. **A)** Siderophore BGC candidates identified by antiSMASH 7.0^21^ on whole genome sequence data for CNSA002. **B)** O-CAS agar assay of CNSA002 colony. **C)** O-CAS agar assay of CNSA002 protein (retentate) and small molecule (flow through) fractions generated using a 3 kDa molecular weight cut off (MWCO) filter.	9](#_Toc237877556)

[**Figure S3.** Most abundant MassQL query hits for **A)** cluster 1: phenol thiazoline, 2-hydroxy-5-oxoproline, *N*-formyl-*N*-hydroxy-ornithine and β-hydroxyasparagine and **B)** clusters 2 and 3: dihydroxybenzoic acid, catechol methyloxazoline, graminine, and citrate.	9](#_Toc237877557)

[**Figure S4.** Aryl thiazoli(di)ne/oxazoli(di)ne siderophores compared in BGC synteny analysis. O and S atoms are circled, differentiating thiazoli(di)nes from oxazoli(di)nes.	10](#_Toc237877558)

[**Figure S5.** Phylogenetic tree depicting BbchE homologous proteins. *Microbulbifer* sp., *Sorangium* sp. and *Luteibacter* sp. clades are pointed out.	10](#_Toc237877559)

[**Figure S6.** TE domain alignment for bulbichelin, sorangibactin, anthrochelin, coelibactin and other TE sequences from PDB. Query for catalytic motif (GxSxG) is highlighted. Bulbichelin, sorangibactin, anthrochelin and coelibactin feature a Cys instead of the common Ser, which is highlighted.	11](#_Toc237877560)

[**Figure S7.** Sequence similarity analysis of BbchB, AntL and SncF along ThYme database thioesterases with known crystal structures. The ThYme database groups thioesterases based on sequence similarity, resulting in 35 thioesterase families (TE1-35)^31^. Here, distinct families are shaded in grey, with family TE18 shaded in red. The localization of BbchB, SncF and AntL in the tree is pointed out. Enzyme classification allows structure and function prediction of uncharacterized enzymes^31^.	12](#_Toc237877561)

[**Figure S8.** Comparison between bulbichelin, sorangibactin and anthrochelin analogs	13](#_Toc237877562)

[**Figure S9.** The structure of bisbulbichelin, confirmed through 1D and 2D NMR.	13](#_Toc237877563)

[**Figure S10.** The ^1^H NMR spectrum of bisbulbichelin (700 MHz, DMSO-*d*_6_)	14](#_Toc237877564)

[**Figure S11.** The ^13^C NMR spectrum of bisbulbichelin (176 MHz, DMSO-*d*_6_)	15](#_Toc237877565)

[**Figure S12.** The HSQC spectrum of bisbulbichelin (700 MHz, DMSO-*d*_6_)	16](#_Toc237877566)

[**Figure S13.** The HMBC spectrum of bisbulbichelin (700 MHz, DMSO-d6)	17](#_Toc237877567)

[**Figure S14.** Mass shifts, isotopic envelopes and absorbance spectra for **A)** Fe(III)-bulbichelin A and **B)** Fe(III)-bisbulbichelin	18](#_Toc237877568)

[**Figure S15.** Representative bulbichelin-Cu infusion replicate 1 isotopic peak patterns around *m/z* 1207 and 604 along with predicted isotopic peak patterns for bisbulbichelin [M + 2Cu(I) – H]^+^, bisbulbichelin [M + 2Cu(I)]^2+^, and bulbichelin C [M + Cu(II) – H] respectively^38^.	20](#_Toc237877569)

[**Figure S16.** Extracted ion chromatogram for feature of interest at 873.497 *m/z* and UV chromatograms for wavelengths 294 nm and 255 nm.	21](#_Toc237877570)

[**Figure S17.** Representative classical networking reanalysis of MSV000084873. MassQL search hits are highlighted in red, including the spectral match for petrobactin.	22](#_Toc237877571)

[**Figure S18.** MS^2^ annotation of fragment peaks for acyl petrobactin C13:1.	23](#_Toc237877572)

[**Figure S19.** MS^2^ spectral analysis of acyl petrobactin, acyl dehydroxypetrobactin and hydroxyacyl petrobactin analogs, compared to petrobactin. Mass shifts for fragments containing catechol are shown in red whereas mass shits for fragments containing phenol are shown in purple. Conserved peaks for both suites are shaded accordingly.	27](#_Toc237877573)

[**Figure S20.** The structure of acyl petrobactin C10:0, elucidated through 1D and 2D NMR.	28](#_Toc237877574)

[**Figure S21.** The ^1^H NMR spectrum of acyl petrobactin C10:0 (800 MHz, DMSO-*d*_6_)	28](#_Toc237877575)

[**Figure S22.** The HSQC spectrum of acyl petrobactin C10:0 (800 MHz, DMSO-*d*_6_)	29](#_Toc237877576)

[**Figure S23.** The HMBC spectrum of acyl petrobactin C10:0 (800 MHz, DMSO-*d*_6_). HMBC stacked comparison for petrobactin and acyl petrobactin, showing additional amide carbonyl correlations with acyl tail hydrogens.	30](#_Toc237877577)

[**Figure S24.** The ^1^H-^1^H TOCSY spectrum of acyl petrobactin C10:0 (800 MHz, DMSO-*d*_6_)	31](#_Toc237877578)

[**Figure S25.** The ^1^H-^1^H NOESY spectrum of acyl petrobactin C10:0 (800 MHz, DMSO-*d*_6_)	32](#_Toc237877579)

[**Figure S26.** Examples of acylated siderophores, acylation is shown in orange	33](#_Toc237877580)

[**Figure S27.** Representative percent adduct calculations for a selection of acyl petrobactin analogs	34](#_Toc237877581)

[**Figure S28.** Petrobactin and roseobactin structural comparison	34](#_Toc237877582)

[**Figure S29.** **A)** O-CAS assay for *Microbulbifer* sp. CNSA002 and *V. coralliilyticus* Cn52-H1 on IDSM media and **B)** OCAS overlay	35](#_Toc237877583)

[**Table S1.** Common siderophore substructures and observed MS^2^ fragments and neutral losses used for MassQL search	36](#_Toc237877584)

[**Table S2.** MASSQL queries utilized to identify potential siderophore features.	41](#_Toc237877585)

[**Table S3.** ^1^H (700 MHz) and ^13^C (175 MHz) NMR data of bisbulbichelin in DMSO-*d*_6_ (*J* in Hz, *δ* in ppm)	43](#_Toc237877586)

[**Table S4.** ^1^H (800 MHz) and ^13^C (200 MHz) NMR data of Acyl petrobactin C10:0 in DMSO-*d*_6_ (*J* in Hz, *δ* in ppm)	44](#_Toc237877587)

[**Table S5.** ^1^H (800 MHz) and ^13^C (200 MHz) NMR data of petrobactin in DMSO-*d*_6_ (*J* in Hz, *δ* in ppm)	45](#_Toc237877588)

[**Table S6.** Putative iron acquisition and transport-related proteins found in *Microbulbifer* sp. CNSA002	46](#_Toc237877589)

[**Table S7.** *Microbulbifer* sp. CNSA002 NRPS siderophore biosynthetic gene cluster proteins predicted function	47](#_Toc237877590)

[**Table S8.** Petrobactin analogs detected in *Microbulbifer* sp. CNSA002 culture and datasets mined with MASST	48](#_Toc237877591)

[**Table S9.** Phylogeny and coassociation analysis of IucA/IucC in *Microbulbifer* sp. CNSA002	49](#_Toc237877592)

[**Table S10.** *Microbulbifer* sp. CNSA002 NI-siderophore biosynthetic gene cluster proteins predicted function	49](#_Toc237877593)

[**Table S11.** Theoretical m/z values for H-bound and all metal-bound acyl petrobactin and bulbichelin variants.	50](#_Toc237877594)

[**Table S12.** Experimental m/z values for H-bound and all metal-bound acyl petrobactin and bulbichelin variants from direct-infusion untargeted LC-MS/MS.	52](#_Toc237877595)

[**Table S13.** Experimental rt values for H-bound and all metal-bound petrobactin and bulbichelin variants from direct infusion untargeted LC-MS/MS.	54](#_Toc237877596)

[**Table S14.** Experimental H-bound peak area and % adduct values for all metal-bound petrobactin and bulbichelin variants from direct infusion untargeted LC-MS/MS.	56](#_Toc237877597)

**Discussion entailing false positives in the output of MassQL**

The identification of *Microbulbifer* sp. CNSA002 metallophores, bulbichelins and acyl petrobactins, exemplifies the value of MassQL for substructure search. However, the data mining conducted simultaneously highlights the need for careful validation of the MassQL output. As mentioned in the main text, the bulbichelin cluster had hits to the phenol thiazoline, 2-hydroxy-5-oxoproline, *N*-formyl-*N*-hydroxy-ornithine and β-hydroxyasparagine queries, despite only incorporating the first of these substructures. On the other hand, the acyl petrobactin features had hits to the dihydroxybenzoic acid, graminine, catechol methyloxazoline, citrate and several hydroxamate queries. These compounds indeed incorporate the dihydroxybenzoic acid and citrate substructures however, they do not contain graminine, catechol methyloxazoline or hydroxamates substructures. These erroneous hits to MassQL queries represent false positives. The MassQL queries included in this study were written to search for an *m/z* within a tolerance, giving leeway for the identification of fragment peaks with different exact masses, and therefore different molecular formulas. Additionally, MassQL queries can identify fragment peaks that match the intended molecular formula but not the intended substructure (i.e. different connectivity of the atoms). For instance, the *m/z* corresponding to the graminine and catechol methyloxazoline queries was indeed observed as fragment peaks in the acyl petrobactins MS^2^ (194.081 and 178.086 *m/z*). However, these peaks correspond to different substructures in the acyl petrobactins (fragmentation is annotated in **Figure S18**). False positives are also exemplified by the identification of ornithine lipids due to the characteristic headgroup fragment coinciding with the *N*-acetyl-*N*-hydroxy-aminopropane query. This demonstrated that, despite searching for siderophore substructures, not all extracted features will correspond to this compound class. These observations evidence the need for detailed scrutiny of MassQL results and manual annotation of extracted features.

**Supplemental Methods**

**Bacterial culturing for untargeted metabolomics**

Bacterial culturing, both in mono- and coculture was previously described^1^. Briefly, strains were inoculated in SWB (saltwater broth) with or without the addition of ethylenediamine-*N*,*N’*-diacetic acid (EDDA, Sigma-Aldrich, St. Louis, MO, USA) (300, 500, 750, 1000 µM) to an OD_600_ of 0.05, using a 1:1 ratio for bacterial cocultures. The cultures were incubated at 30 °C, shaking at 200 rpm for 24 h. Harvested cultures were extracted using a variety of techniques: liquid-liquid extraction (LLE) with ethyl acetate (EtOAc), solid phase extraction (SPE) with C18 columns (Thermo Scientific, Waltham, MA) and solid liquid extraction (SLE) with 2:2:1 ethyl acetate:methanol:water (EtOAc:MeOH:H_2_O). Extracts were dried *in vacuo* and stored at −20 °C until data acquisition.

**Untargeted metabolomics data acquisition and analysis**

The dried extracts were resuspended in 300 µL of 100% MeOH containing 1 µM of sulfadimethoxine as an internal standard. Samples were vortexed, sonicated for 10 min and centrifuged at 16,160 x g for 15 min. The resuspended extracts were analyzed using an Agilent 1290 Infinity II Ultra High-Pressure Liquid Chromatography (UHPLC) system (Agilent Technologies; Santa Clara, CA, USA) with a Kinetex 1.7 μm C18 reversed phase UHPLC column (50 × 2.1 mm) (Phenomenex; Torrance, CA, USA) coupled to an ImpactII ultrahigh resolution Qq-ToF mass spectrometer (Bruker Daltonics, GmbH, Bremen, Germany) equipped with an electrospray ionization (ESI) source. Chromatographic separation was performed with the following mobile phase gradient: 5% solvent B (MeCN, 0.1% (v/v) formic acid) and 95% solvent A (H_2_O, 0.1% (v/v) formic acid) for 3 min, a linear gradient of 5% B–95% B over 17 min, held at 95% B for 3 min, 95% B–5% B in 1 min, and held at 5% B for 1 min, 5% B- 95% B in 1 min, held at 95% B for 2 min, 95% B−5% B in 1 min, and held at 5% B for 2.5 min, at a flow rate of 0.5 mL/min throughout. MS spectra were acquired in positive ionization mode from *m/z* 50 to 2000 Da. An external calibration with ESI-L Low Concentration Tuning Mix (Agilent Technologies) was performed prior to data collection, and hexakis(1H,1H,2H-perfluoroethoxy)phosphazene was used as an internal lock-mass calibrant throughout the run. The eight most intense ions per MS^1^ were selected for fragmentation and MS^2^ data acquisition. A basic stepping function was used to fragment ions at 50% and 125% of the CID calculated for each *m/z* with timing of 50% for each step. The MS/MS active exclusion parameter was set to two, and the active exclusion was released after 30s. The mass of the internal lock-mass calibrant was excluded from the MS^2^ list. UV data was acquired with a UV DAD detector (Agilent Technologies) from 190 nm to 400 nm, with a 2 nm step. Zero offset was set at 5% along a 1000 mAU attenuation. Data was acquired throughout the LC run with a >0.1 min peak width.

Raw data were converted to mzXML format, using vendor proprietary software. Metabolite features were extracted using MZmine 2.53^2^, performing mass detection, chromatogram building, chromatogram deconvolution, isotopic peak grouping, retention time alignment, replicate filtering, duplicate peak removal, and gap filling. The resulting processed data was submitted to the Global Natural Product Social Molecular Network platform (GNPS) to generate a feature-based molecular network (FBMN). The molecular network was generated using the following parameters: fragment ions were removed within a ±17 Da window of the precursor *m/z*, precursor ion and fragment ion mass tolerance were set to 0.02 Da and edges were filtered to have a score above 0.7 and at least 4 matched peaks. Edges were kept if both nodes were present in each other’s top 10 most similar nodes, and molecular families’ maximum size was set to 100. Experimental fragmentation spectra were searched against GNPS’s spectral libraries and filtered in the same way (cosine score above 0.7 and a minimum of 4 matched peaks). The workflows for DEREPLICATOR^3^, DEREPLICATOR+^4^, MolDiscovery^5^ and MS2LDA^6^ were also run on GNPS. The output from MZmine was additionally exported for analysis with SIRIUS^7^ 5.3.6. with CSI:FingerID^8^ and CANOPUS^9^. These tools provide putative annotations, which are confirmed using an MS^2^ spectral comparison with literature reported spectra, in-house spectra, with data acquired on analytical standards, and manual annotation of MS^2^ fragments resulting in level 2 compound annotations. SIRIUS computes putative chemical formulas using MS^1^ and fragmentation trees (based on user uploaded MS^1^ isotopic peaks and MS^2^ fragmentation patterns). CSI: FingerID transforms MS^2^ spectra into predicted structural fingerprints that enable matching to chemical databases.  CANOPUS predicts the chemical class of metabolites by utilizing CSI:FingerID's predicted structural fingerprints. Library and database searches were performed for The Natural Product Atlas^10^, LOTUS^11^, MarinLit^12^, Pubchem and Scifinder. The molecular network was visualized using Cytoscape^13^ v3.9.0 and features present in the blanks were subtracted.

A MassQL search was conducted in GNPS for each of the 30 siderophore substructures (**Table S1**). These queries search for common MS^2^ peaks and neutral losses detected for siderophores including each substructure and delimits the *m/z* search to a 10 ppm window and 20% intensity threshold (**Table S2**). The results from each query were extracted and visualized using the classical networking workflow in GNPS.

Regarding cluster 1, a MASST search was conducted in GNPS for the 525.112 *m/z* ion, however, no hits were retrieved. On the other hand, for clusters 2 and 3, a search was conducted for five parent ions (761.371, 873.497, 887.511, 899.513, and 913.528 *m/z*), and five fragment peaks (194.081, 282.181, 324.192, 396.176, and 438.186 *m/z*) shared by the features of interest. Two datasets (MSV000088745 and MSV000084873) contained features matching our search and they were reanalyzed, generating classical networks in order to identify related features.

**Fermentation, extraction, and compound purification of bisbulbichelin**

*Microbulbifer* sp. CNSA002 was inoculated at an OD_600_ of 0.05 in 2.8 L baffled flasks containing 1 L of marine broth at half concentration of nutrients (MB/2) supplemented with 750 µM EDDA. Cultures were incubated at 30 °C, shaking at 180 rpm for 24 h. Harvested cultures (8 L) were extracted two times with ethyl acetate. Extracts were combined and the solvent was dried *in vacuo* to yield a 0.5 g crude extract.

The crude extract was resuspended in MeOH and fractionated using an Agilent 1260 Infinity III Liquid Chromatography (semipreparative HPLC) system (Agilent Technologies) equipped with an Eclipse XBD-C18 5 μm reversed phase HPLC column (250 × 9.4 mm) (Agilent Technologies). The mobile phases used for chromatographic separation were solvent A: H_2_O, 0.1% (*v/v*) formic acid (FA) and solvent B: MeCN, 0.1% (*v/v*) FA. The gradient was performed with the following mobile phase compositions: a linear gradient from 5% solvent B to 100% solvent B in 25 min, held at 100% B for 10 min, at a flow rate of 2 mL/min throughout. Elution was performed with a UV detector monitoring the run at 198 nm, 250 nm, and 352 nm. Fraction 1 contained a mixture of Fe-bound and apo bulbichelin A, fraction 3 contained a mixture of Fe-bound and apo bulbichelin A, as well as bulbichelin C. Fraction 4 contained bisbulbichelin (compound **1**) (1.0 mg).

Compound **1**: white powder. ^1^H and ^13^C NMR data, see **Table S3**. Positive HRESIMS *m/z* 1083.220 [M+H]^+^ (calculated for C_44_H_59_N_8_O_8_S_8_^+^, 1083.2217)

One-dimensional (1D) and two-dimensional (2D) NMR spectra were recorded on a Bruker AV3 HD 700 MHz NMR, in DMSO-*d*_6_ and calibrated using residual undeuterated solvent as internal reference. The chemical shift (*δ*) is reported in parts per million (ppm) and the coupling constants (*J* values) are in Hz. NMR data for compound **1** has been deposited to NP-MRD^14^ (ID: NP0352519).

**Fermentation, extraction, and compound purification of acyl petrobactins**

*Microbulbifer* sp. CNSA002 was inoculated at an OD_600_ of 0.05 in 1 L flasks containing 200 mL of SWB. Cultures were incubated at 30 °C, shaking at 200 rpm for 24 h. Harvested cultures (7 L) were centrifuged at 4920 × g for 45 min at 4 °C to separate the cell pellet from supernatant. The cell-free supernatant was mixed with 4% (*w/v*) XAD-16N resin (Sigma-Aldrich, St. Louis, MO, USA) and stirred for 16 h. Afterwards, the supernatant was removed, and the resin was washed with 500 mL of H_2_O and eluted with 500 mL of ethanol (EtOH). The cell pellet was disrupted using a tissue homogenizer (Tissue Master 125, Omni International, Kennesaw, GA, USA) and extracted with 50 mL of 2:2:1 EtOAc:MeOH:H_2_O. The extraction was vortexed every 30 min for 8 h. Both extracts were combined and the solvent was dried *in vacuo* to yield a 5 g crude extract.

The crude extract was resuspended in H_2_O and fractionated *via* reverse phase liquid chromatography (RPLC) using a 10 g C18 column (Thermo Scientific, Waltham, MA). The column was washed with 150 mL of MeCN and equilibrated with 150 mL of H_2_O. The crude was loaded, and the analytes were eluted with 20%, 40% and 100% MeCN to obtain three fractions (1-3) which were dried *in vacuo*. Fraction 1 (20% MeCN) was further fractionated using an Agilent 1260 Infinity II Liquid Chromatography (semipreparative HPLC) system (Agilent Technologies) equipped with a Luna 5 μm C18 reversed phase HPLC column (250 × 10 mm) (Phenomenex). The mobile phases used for chromatographic separation were solvent A: H_2_O, 0.1% (*v/v*) TFA and solvent B: MeCN, 0.1% (*v/v*) trifluoroacetic acid (TFA). The gradient was performed with the following mobile phase compositions: a linear gradient from 5% solvent B to 100% solvent B in 15 min, held at 100% B for 10 min, at a flow rate of 2 mL/min throughout. Elution was performed with a UV detector monitoring the run at 190 nm and 254 nm. Fraction 7 (10.2 mg) contained petrobactin and fraction 14 (17.2 mg) contained a mixture of acylated petrobactins. Fraction 7 was further fractionated using the following mobile phase gradient: a linear gradient from 5% solvent B to 100% solvent B in 25 min, held at 100% B for 10 min, at a flow rate of 2 mL/min throughout. This fractionation yielded 5.6 mg of petrobactin. Fraction 14 was further fractionated using the following mobile phase gradient: a linear gradient from 5% solvent B to 60% solvent B in 80 min, 60% solvent B to 100% solvent B in 5 min, held at 100% B for 15 min, at a flow rate of 2 mL/min throughout. This fractionation yielded compound **2** (F14.14) (1.0 mg) and two mixed fractions: F14.15 and F14.16 (2.6 mg and 1.0 mg).

Compound **2**: yellow, oily. ^1^H and ^13^C NMR data, see **Table S4**. Positive HRESIMS *m/z* 873.497 [M+H]^+^ (calculated for C_44_H_69_N_6_O_12_^+^, 873.4968)

Petrobactin: yellow, oily. ^1^H and ^13^C NMR data, see **Table S5**. Positive HRESIMS *m/z* 719.361 [M+H]^+^ (calculated for C_34_H_51_N_6_O_11_^+^, 719.3610)

1D and 2D NMR spectra were recorded on a Bruker Avance III HD 800 MHz NMR, in DMSO-*d*_6_ and calibrated using residual undeuterated solvent as internal reference. The chemical shift (*δ*) is reported in parts per million (ppm) and the coupling constants (*J* values) are in Hz. NMR data for compound **2** has been deposited to NP-MRD^14^ (ID: NP0351565).

**O-CAS agar assay**

A modified CAS assay (O-CAS^15^) was overlaid on a colony of *Microbulbifer* sp. CNSA002 grown on SWA. The spent SWB media of *Microbulbifer* sp. CNSA002 was fractionated using a molecular weight cutoff (MWCO) Amicon ultracentrifugation filter (3 kDa, Sigma-Aldrich) and each fraction was tested on an O-CAS agar plate. The iron chelating activity of compound **2** and fractions F14.15 and F14.16 were also tested on an O-CAS plate. A 2.5 µL aliquot of each a 200 mg/mL methanolic solution of compound **2**, 400 mg/mL F14.15 and 200 mg/mL F14.16 were spotted on an O-CAS agar plate^15^. Desferrioxamine mesylate (Sigma-Aldrich) 1 mg/mL was used as a positive control, whereas methanol was spotted as a negative control. The plates were incubated at room temperature overnight before registering the results.

*Microbulbifer* sp. CNSA002 and *V. coralliilyticus* Cn52-H1 overnight cultures were spotted on IDSM^16^ agar, either as single spots or adjacent spots. Plates were incubated at 30 °C for 5 days prior to O-CAS overlay, the plates were incubated at room temperature overnight before registering the results.

**CNSA002 whole genome sequencing and analysis**

We had previously sequenced the *Microbulbifer* sp. CNSA002 genome using Illumina and Nanopore platforms, and deposited to NCBI under the bioproject PRJNA1173768 (NCBI:txid3373604). The genome was analyzed using the PhylaLite and TypeMat workflows under the MiGA platform^17^. Genome annotation was performed in the RAST platform^18^ using the RASTtk^19^ scheme and browsed with SEED viewer^20^. Additional annotation of biosynthetic gene clusters was performed in the bacterial version of antiSMASH^21^ (version 7.0) with a relaxed strictness, and NCBI PGAP^22^. AntiSMASH predicted an NRPS metallophore and an NI-siderophore BGC, whereas RAST-SEED predicted the latter to be for anthrachelin biosynthesis, a purported NI-siderophore.

We used the CAGECAT^23^ clinker tool to compare the NRPS siderophore BGC from *Microbulbifer* sp. CNSA002 and known aryl thiazoli(di)ne/oxazoli(di)ne siderophore BGCs from MIBiG^24^: sorangibactin (BGC0002912), coelibactin (BGC0000324), ulbactin (BGC0002472), thiazostatin (BGC0001801), enantio-pyochelin (BGC0002475), yersiniabactin (BGC0001055), piscibactin (BGC0002533). *Microbulbifer* sp. CNSA002 NRPS BGC contained two core biosynthetic genes, *bbchE* and *bbchF.* Homologous proteins to BbchE were searched for using the blastp function in NCBI’s BLAST^25^. The 100 most similar sequences were downloaded and aligned using ClustalW^26^ in MEGA11^27^. The aligned sequences were used to generate a phylogenetic tree using the maximum likelihood method and JTT matrix-based model^28^. The tree was visualized in iTOL^29^. BbchF TE domain was compared through multiple sequence alignment with other TE domains, including the sorangibactin and coelibactin thioesterases using MUSCLE^30^ in MEGA11. Thioesterase phylogenetic analysis was performed by downloading sequences of TEs with known protein crystal structure in the ThYme database^31^ (as of February 2026). Sequences for BbchB, AntL and SncF were also added to the analysis . All sequences were aligned using MUSCLE^30^, and a phylogenetic tree was generated in MEGA11 using a JTT matrix-based model^28^. Family nomenclature was assigned based on the ThYme database.

The NIS BGC contained two *iucA/iucC* genes which were input into EFI-EST^32, 33^ (enzyme function initiative-enzyme similarity tool) for analysis using the default parameters. The SSN alignment score threshold was set to 40%. The representative SSN (40% identity) containing 216 nodes was exported and visualized in Cytoscape. The corresponding table was exported as a csv and the accession numbers were used to obtain the RefSeq sequences using the UniProt^34^ ID Map. Protein sequences were retrieved using the NCBI Batch Entrez, non-redundant sequences (139) were aligned using ClustalW^26^ in the CIPRES science gateway^35^. A tree was constructed using the RaxML-HPC Blackbox^36^ workflow using default parameters. The program determined 350 bootstraps to be sufficient, and the output tree was visualized in iTOL^29^. The clade including *Microbulbifer* sp. CNSA002 was identified and selected for further analysis. In order to identify co-occurring genes, the representative SSN was submitted to the enzyme function initiative genome neighborhood tool (EFI-GNT)^33^. The neighborhood size was set to 15 and the minimal co-occurrence limit was set to 20%. The results were visualized in Cytoscape and the respective table was exported. The IucA/IucC entries belonging to the clade of interest were identified and visualized on the genome neighborhood diagram, where co-occurring Pfam families were identified. Protein co-occurrence data was converted into binary annotation and mapped onto the tree using iTOL.

Given the similarity to the petrobactin BGC and the presence of a petrobactin feature in the MSV000084873 dataset with a very similar fragmentation pattern, we used the CAGECAT^23^ clinker tool to compare the NI-siderophore BGC from *Microbulbifer* sp. CNSA002 and published petrobactin BGCs from different bacteria. *B. anthracis* Ames strain petrobactin cluster was obtained from MIBiG^24^ (BGC0000942). The biosynthetic gene clusters for *A. macleodii* ATCC 27126 (NCBI:txid529120) and *M. nauticus* VT8 (NCBI:txid351348, heterotypic synonym *M. hydrocarbonoclasticus*) were obtained by analyzing the whole genome sequences on antiSMASH. Similarly, the BGCs for the structurally related rhodopetrobactin and methylolanthanin were obtained from the genomes of *R. palustris* TIE-1 (NCBI:txid395960) and *M. extorquens* AM1 (NCBI:txid272630). Additionally, genes in the petrobactin cluster were queried against *Microbulbifer* sp. CNSA002 using the tblastn functionality in NCBI’s BLAST^25^ (**Table S10**). BGC boundaries were determined using RAST-SEED predictions.

**Direct metal infusion on UHPLC-MS/MS and data analysis**

Relative binding assays were analyzed using a Thermo Scientific Q Exactive HF with upstream Vanquish UHPLC with a Kinetex 1.7 μm EVO C18 column (50 x 1.0 mm, 100 Å pore size) (Phenomenex; Torrance, CA, USA). A 5 µL injection was subjected to chromatographic separation at a flow rate of 0.150 mL/min with post-column metal infusion at 0.005 mL/min using a syringe pump. A 0.150 mL/min 10 mM ammonium acetate stream was fed using a PEEK T-splitter post-LC to keep the resulting stream at a pH ~6^37^. Chromatographic separation was performed with the following mobile phases and gradient: A (H_2_O + 0.1% (v/v) formic acid) and B (acetonitrile + 0.1% (v/v) formic acid) with a linear gradient from 2.0 % B to 99.0% B over 6 min, held for 2 min as a washout, re-equilibrated by returning to 2.0 % B by 8.1 min, and held until 12 min. MS spectra were acquired in positive ionization mode from *m/z* 350-1500 Da for petrobactin and derivatives and 250-1500 Da for bulbichelin and derivatives (in order to observe the [M+2H]^2+^of Bulbichelin C) to 1500 Da (MS1 resolution of 45,000) and 100 ms maximum injection time with an AGC target of 1E6. The five most intense ions per MS^1^ were selected for fragmentation and MS^2^ data acquisition with a resolution of 15,000, and isolation window of 1.0 m/z. A stepped normalized collision energy of 25, 35, and 45 eV was used for fragmentation. Intensity threshold for selected ions was set to 1.6E5 and precursors were selected using an apex trigger of 2 to 15 s from first detection. The MS/MS dynamic exclusion parameter was set to 5 s. MS^2^ maximum injection time was 50 ms with an AGC target of 5E5 and minimum of 8E3. All data were acquired with the following parameters: sheath gas flow of 35 arbitrary units (AU), auxiliary gas flow of 10 AU, and sweep gas flow of 1 AU. Additionally, the auxiliary gas heater temperature was 200 °C, spray voltage was 3.5 kV, the capillary temperature was 250 °C, and the S-lens RF level was set to 55 AU. Metal solutions for infusion were prepared fresh on the day of use with LCMS-grade water and filtered prior to loading into the syringe. FeCl_3_ was used to provide Fe^3+^ ions, ZnCl_2_ for Zn^2+^ ions, CoCl₂·6H₂O for Co^2+^ ions, MnCl_2_·4H_2_O for Mn^2+^ ions, NiCl_2_·6H_2_O for Ni^2+^ ions, and (where applicable) CuSO_4_·5H_2_O for Cu^2+^ ions. All metal solutions were prepared at concentrations of 2 and (where applicable) 0.5 mM.

Raw data files were converted to the mzML format using msConvert by Proteowizard as previously described^37^. MS data was processed with mzmine 4.7.29 to produce feature tables from the data files. Peak-picking was performed with *m/z* 10 ppm tolerance and retention time with 0.16 min tolerance. A table of calculated and found *m/z* and retention time for each [M+H]^+^ and metal-adduct was prepared from the original feature table (**Tables S11-13**). A binding percentage, calculated as the metal-adduct peak area divided by the total peak area of the metal adduct plus the [M+H]^+^ adduct in each infusion run, was calculated for each infused metal and concentration (**Table S14**). Metal-adducts were not searched for if [M+H]^+^ species was not found. Bar graphs were prepared using all replicates (n = 3) for each infusion using the Plotly package for python with standard deviations shown as error bars. All files relating to this analysis (e.g., mzML, mzmine batch, python code, excel) can be found in deposited on MassIVE or in Github.

**Figure S1.** Examples of α-hydroxycarboxylate, catechol and hydroxamate siderophores. Iron-chelating moieties are circled. Mixed siderophores, incorporating more than one moiety, are presented as well.

**Figure S2.** Production of siderophores by *Microbulbifer* sp. CNSA002. **A)** Siderophore BGC candidates identified by antiSMASH 7.0^21^ on whole genome sequence data for CNSA002. **B)** O-CAS agar assay of CNSA002 colony. **C)** O-CAS agar assay of CNSA002 protein (retentate) and small molecule (flow through) fractions generated using a 3 kDa molecular weight cut off (MWCO) filter.

**Figure S3.** Most abundant MassQL query hits for **A)** cluster 1: phenol thiazoline, 2-hydroxy-5-oxoproline, *N*-formyl-*N*-hydroxy-ornithine and β-hydroxyasparagine and **B)** clusters 2 and 3: dihydroxybenzoic acid, catechol methyloxazoline, graminine, and citrate.

**Figure S4.** Aryl thiazoli(di)ne/oxazoli(di)ne siderophores compared in BGC synteny analysis. O and S atoms are circled, differentiating thiazoli(di)nes from oxazoli(di)nes.

**Figure S5.** Phylogenetic tree depicting BbchE homologous proteins. *Microbulbifer* sp., *Sorangium* sp. and *Luteibacter* sp. clades are pointed out.

**Figure S6.** TE domain alignment for bulbichelin, sorangibactin, anthrochelin, coelibactin and other TE sequences from PDB. Query for catalytic motif (GxSxG) is highlighted. Bulbichelin, sorangibactin, anthrochelin and coelibactin feature a Cys instead of the common Ser, which is highlighted.

**Figure S7.** Sequence similarity analysis of BbchB, AntL and SncF along ThYme database thioesterases with known crystal structures. The ThYme database groups thioesterases based on sequence similarity, resulting in 35 thioesterase families (TE1-35)^31^. Here, distinct families are shaded in grey, with family TE18 shaded in red. The localization of BbchB, SncF and AntL in the tree is pointed out. Enzyme classification allows structure and function prediction of uncharacterized enzymes^31^.

**Figure S8.** Comparison between bulbichelin, sorangibactin and anthrochelin analogs

**Figure S9.** The structure of bisbulbichelin, confirmed through 1D and 2D NMR.

**Figure S10.** The ^1^H NMR spectrum of bisbulbichelin (700 MHz, DMSO-*d*_6_)

**Figure S11.** The ^13^C NMR spectrum of bisbulbichelin (176 MHz, DMSO-*d*_6_)

**Figure S12.** The HSQC spectrum of bisbulbichelin (700 MHz, DMSO-*d*_6_)

**Figure S13.** The HMBC spectrum of bisbulbichelin (700 MHz, DMSO-d6)

**Figure S14.** Mass shifts, isotopic envelopes and absorbance spectra for **A)** Fe(III)-bulbichelin A and **B)** Fe(III)-bisbulbichelin

**Figure S15.** Representative bulbichelin-Cu infusion replicate 1 isotopic peak patterns around *m/z* 1207 and 604 along with predicted isotopic peak patterns for bisbulbichelin [M + 2Cu(I) – H]^+^, bisbulbichelin [M + 2Cu(I)]^2+^, and bulbichelin C [M + Cu(II) – H] respectively^38^.

**Figure S16.** Extracted ion chromatogram for feature of interest at 873.497 *m/z* and UV chromatograms for wavelengths 294 nm and 255 nm.

**Figure S17.** Representative classical networking reanalysis of MSV000084873. MassQL search hits are highlighted in red, including the spectral match for petrobactin.

**Figure S18.** MS^2^ annotation of fragment peaks for acyl petrobactin C13:1.

**Figure S19.** MS^2^ spectral analysis of acyl petrobactin, acyl dehydroxypetrobactin and hydroxyacyl petrobactin analogs, compared to petrobactin. Mass shifts for fragments containing catechol are shown in red whereas mass shits for fragments containing phenol are shown in purple. Conserved peaks for both suites are shaded accordingly.

**Figure S20.** The structure of acyl petrobactin C10:0, elucidated through 1D and 2D NMR.

**Figure S21.** The ^1^H NMR spectrum of acyl petrobactin C10:0 (800 MHz, DMSO-*d*_6_)

**Figure S22.** The HSQC spectrum of acyl petrobactin C10:0 (800 MHz, DMSO-*d*_6_)

**Figure S23.** The HMBC spectrum of acyl petrobactin C10:0 (800 MHz, DMSO-*d*_6_). HMBC stacked comparison for petrobactin and acyl petrobactin, showing additional amide carbonyl correlations with acyl tail hydrogens.

**Figure S24.** The ^1^H-^1^H TOCSY spectrum of acyl petrobactin C10:0 (800 MHz, DMSO-*d*_6_)

**Figure S25.** The ^1^H-^1^H NOESY spectrum of acyl petrobactin C10:0 (800 MHz, DMSO-*d*_6_)

**Figure S26.** Examples of acylated siderophores, acylation is shown in orange

**Figure S27.** Representative percent adduct calculations for a selection of acyl petrobactin analogs

**Figure S28.** Petrobactin and roseobactin structural comparison

**Figure S29.** **A)** O-CAS assay for *Microbulbifer* sp. CNSA002 and *V. coralliilyticus* Cn52-H1 on IDSM media and **B)** OCAS overlay

**Table S1.** Common siderophore substructures and observed MS^2^ fragments and neutral losses used for MassQL search

| Ion | *m/z* fragment | Neutral loss | Siderophore family | Fragment | Examples |
| --- | --- | --- | --- | --- | --- |
| β-hydroxyaspartate | 132.0291 | 131.0219 | α-hydroxycarboxylate |  | Aquachelin, marinobactin, alterobactin, ornibactin |
| β-hydroxyaspartate | 150.0397 | 149.0324 | α-hydroxycarboxylate |  | Aquachelin, marinobactin, alterobactin, ornibactin |
| β-hydroxyhistidine | 154.0611 | 153.0538 | α-hydroxycarboxylate |  | Histicorrugatin |
| β-hydroxyhistidine (terminal) | 172.0717 | 171.0644 | α-hydroxycarboxylate |  | Histicorrugatin |
| β-hydroxyasparagine | 131.0451 | 130.0378 | α-hydroxycarboxylate |  | Pyoverdine VLB120 |
| β-hydroxyasparagine | 149.0557 | 148.0484 | α-hydroxycarboxylate |  | Pyoverdine VLB120 |
| Hydroxysuccinate | 117.0182 | 116.011 | α-hydroxycarboxylate |  | Rhizobactin |
| 2-hydroxy-5-oxoproline | 146.0448 | 145.0375 | α-hydroxycarboxylate |  | Vibrioferrin |
| Citrate |  | 156.0059 | α-hydroxycarboxylate |  | Petrobactin, aerobactin |
| Citrate | 175.0237 | 174.0164 | α-hydroxycarboxylate |  | Petrobactin, aerobactin |
| Dihydroxybenzoic acid (DHBA) | 137.0233 |  | catechol |  | Petrobactin, vanchrobactin, vibriobactin, vulnibactin |
| Ferribactin | 305.1608 |  | catechol |  | Pyoverdine precursors |
| Ferribactin | 170.0924 |  | catechol |  | Pyoverdine precursors |
| Ferribactin | 136.0757 |  | catechol |  | Pyoverdine precursors |
| Pyoverdine chromophore | 204.0768 |  | catechol |  | Pyoverdines |
| Isopyoverdine chromophore | 230.0924 |  | catechol |  | Pyoverdines |
| Graminine | 178.0822 | 177.0750 | diazeniumdiolate |  | Gramibactin, trinickiabactin |
| Graminine | 160.0717 | 159.0644 | diazeniumdiolate |  | Gramibactin, trinickiabactin |
| *N-*hydroxy-*N-*succinyl-cadaverine | 201.1234 |  | hydroxamate |  | Desferrioxamine |
| *N-*hydroxy-*N-*succinyl-cadaverine | 219.1339 |  | hydroxamate |  | Desferrioxamine |
| *N-*hydroxy-*N-*succinyl-cadaverine fragment | 119.1179 |  | hydroxamate |  | Desferrioxamine |
| *N-*hydroxy-*N-*succinyl-cadaverine fragment | 101.0233 |  | hydroxamate |  | Desferrioxamine |
| *N-*acetyl-*N-*hydroxy-cadaverine | 161.1285 | 160.1212 | hydroxamate |  | Desferrioxamine |
| *N-*hydroxy- putrescine | 105.1022 | 104.0950 | hydroxamate |  | Desferrioxamine |
| *N-*hydroxy-*N-*succinyl-putrescine | 187.1077 | 186.1004 | hydroxamate |  | Avaroferrin, putrebactin |
| *N-*acetyl-*N-*hydroxy-lysine | 205.1183 |  | hydroxamate |  | Aerobactin, ochrobactin |
| *N-*acetyl-*N-*hydroxy-lysine | 187.1077 | 186.1004 | hydroxamate |  | Aerobactin, ochrobactin |
| *N-*formyl-*N-*hydroxy-ornithine | 177.0870 | 176.0797 | hydroxamate |  | Lystabactin, ornibactin |
| *N-*formyl-*N-*hydroxy-ornithine | 159.0764 | 158.0691 | hydroxamate |  | Lystabactin, ornibactin |
| *N-*acetyl-*N-*hydroxy-ornithine | 191.1026 | 190.0954 | hydroxamate |  | Amphibactin, marinobactin, moanachelin, loihichelin, aquachelin |
| *N-*acetyl-*N-*hydroxy-ornithine | 173.0921 | 172.0848 | hydroxamate |  | Amphibactin, marinobactin, moanachelin, loihichelin, aquachelin |
| Cyclo *N-*hydroxy-ornithine | 131.0815 | 130.0742 | hydroxamate |  | Serobactin |
| *N-*acetyl-*N-*hydroxy-aminopropane | 133.0972 | 132.0899 | hydroxamate |  | Schizokinen |
| *N-*anhydromevalonyl-*N-*hydroxy-ornithine | 261.1445 | 260.1372 | hydroxamate |  | Fusarinines |
| *N-*hydroxy-*N-*(3-hydroxybutyryl)-ornithine | 235.1288 | 234.1216 | hydroxamate |  | Cupriachelin, ornibactin |
| *N-*hydroxy-histamine | 128.0818 |  | hydroxamate |  | Anguibactin |
| Phenol oxazoline | 164.0706 |  | Mixed thia/oxazoli(di)ne |  | Nigribactin |
| Phenol oxazoline | 190.0499 |  | Mixed thia/oxazoli(di)ne |  | Nigribactin |
| Phenol methyloxazoline | 178.0863 |  | Mixed thia/oxazoli(di)ne |  | Vulnibactin, parabactin |
| Phenol methyloxazoline | 204.0655 |  | Mixed thia/oxazoli(di)ne |  | Vulnibactin, parabactin |
| Phenol thiazoline | 180.0478 |  | Mixed thia/oxazoli(di)ne |  | Pyochelin, piscibactin, ulbactin, yersiniabactin |
| Phenol thiazoline | 190.0321 |  | Mixed thia/oxazoli(di)ne |  | Pyochelin, piscibactin, ulbactin, yersiniabactin |
| Methylthiazolidine carboxylic acid | 146.0270 |  | Mixed thia/oxazoli(di)ne |  | Pyochelin |
| Methylthiazoline carboxylic acid | 144.0114 |  | Mixed thia/oxazoli(di)ne |  | Piscibactin |
| Methylthiazoline carboxylic acid | 160.0426 |  | Mixed thia/oxazoli(di)ne |  | Piscibactin |
| Catechol mehtyloxazoline | 220.0604 |  | Mixed thia/oxazoli(di)ne |  | Vibriobactin, fluvibactin |
| Catechol mehtyloxazoline | 194.0812 |  | Mixed thia/oxazoli(di)ne |  | Vibriobactin, fluvibactin |
| Catechol thiazoline | 222.0219 |  | Mixed thia/oxazoli(di)ne |  | Anguibactin |
| Catechol thiazoline | 194.0270 |  | Mixed thia/oxazoli(di)ne |  | Anguibactin |

**Table S2.** MASSQL queries utilized to identify potential siderophore features.

| Ion | Query |
| --- | --- |
| Iron-bound molecules^39^ | QUERY scaninfo(MS2DATA) WHERE MS1MZ=X-1.993:INTENSITYMATCH=Y*0.063:INTENSITYMATCHPERCENT=25:TOLERANCEPPM=10 AND MS1MZ=X:INTENSITYMATCH=Y:INTENSITYMATCHREFERENCE:INTENSITYPERCENT=5 AND MS1MZ=X+1.0034:TOLERANCEPPM=10 AND MS1MZ=X-52.91:TOLERANCEPPM=10 AND MS2PREC=X-52.91 \|\|\| QUERY scaninfo(MS2DATA) WHERE MS1MZ=X-1.993:INTENSITYMATCH=Y*0.063:INTENSITYMATCHPERCENT=25:TOLERANCEPPM=10 AND MS1MZ=X:INTENSITYMATCH=Y:INTENSITYMATCHREFERENCE:INTENSITYPERCENT=5 AND MS1MZ=X+1.0034:TOLERANCEPPM=10 AND MS1MZ=X-52.91:TOLERANCEPPM=10 AND MS2PREC=X |
| β-hydroxyaspartate | QUERY scaninfo(MS2DATA) WHERE MS2PROD=(132.0291 OR 150.0397):TOLERANCEPPM=10:INTENSITYPERCENT=20\|\|\|QUERY scaninfo(MS2DATA) WHERE MS2PROD=X AND MS2PROD=(X-131.0219):TOLERANCEPPM=10:INTENSITYPERCENT=20\|\|\| QUERY scaninfo(MS2DATA) WHERE MS2PROD=X AND MS2PROD=(X-149.0324):TOLERANCEPPM=10:INTENSITYPERCENT=20 |
| β-hydroxyhistidine | QUERY scaninfo(MS2DATA) WHERE MS2PROD=(154.0611 OR 172.0717):TOLERANCEPPM=10:INTENSITYPERCENT=20\|\|\|QUERY scaninfo(MS2DATA) WHERE MS2PROD=X AND MS2PROD=(X-153.0538):TOLERANCEPPM=10:INTENSITYPERCENT=20\|\|\| QUERY scaninfo(MS2DATA) WHERE MS2PROD=X AND MS2PROD=(X-171.0644):TOLERANCEPPM=10:INTENSITYPERCENT=20 |
| β-hydroxyasparagine | QUERY scaninfo(MS2DATA) WHERE MS2PROD=(131.0451 OR 149.0557):TOLERANCEPPM=10:INTENSITYPERCENT=20\|\|\|QUERY scaninfo(MS2DATA) WHERE MS2PROD=X AND MS2PROD=(X-130.0378):TOLERANCEPPM=10:INTENSITYPERCENT=20\|\|\| QUERY scaninfo(MS2DATA) WHERE MS2PROD=X AND MS2PROD=(X-148.0484):TOLERANCEPPM=10:INTENSITYPERCENT=20 |
| Hydroxysuccinate | QUERY scaninfo(MS2DATA) WHERE MS2PROD=(117.0182):TOLERANCEPPM=10:INTENSITYPERCENT=20\|\|\|QUERY scaninfo(MS2DATA) WHERE MS2PROD=X AND MS2PROD=(X-116.011):TOLERANCEPPM=10:INTENSITYPERCENT=20 |
| 2-hydroxy-5-oxoproline | QUERY scaninfo(MS2DATA) WHERE MS2PROD=(146.0448):TOLERANCEPPM=10:INTENSITYPERCENT=20\|\|\|QUERY scaninfo(MS2DATA) WHERE MS2PROD=X AND MS2PROD=(X-145.0375):TOLERANCEPPM=10:INTENSITYPERCENT=20 |
| Citrate | QUERY scaninfo(MS2DATA) WHERE MS2PROD=(175.0237):TOLERANCEPPM=10:INTENSITYPERCENT=20\|\|\|QUERY scaninfo(MS2DATA) WHERE MS2PROD=X AND MS2PROD=(X-156.0059):TOLERANCEPPM=10:INTENSITYPERCENT=20\|\|\|QUERY scaninfo(MS2DATA) WHERE MS2PROD=X MS2PROD=(X-174.0164):TOLERANCEPPM=10:INTENSITYPERCENT=20 |
| DHBA | QUERY scaninfo(MS2DATA) WHERE MS2PROD=(137.0233):TOLERANCEPPM=10:INTENSITYPERCENT=20 |
| Ferribactin | QUERY scaninfo(MS2DATA) WHERE MS2PROD=(305.1608 OR 170.0924 OR 136.0757):TOLERANCEPPM=10:INTENSITYPERCENT=20 |
| Pyoverdine chromophore | QUERY scaninfo(MS2DATA) WHERE MS2PROD=(204.0768):TOLERANCEPPM=10:INTENSITYPERCENT=20 |
| Isopyoverdine chromophore | QUERY scaninfo(MS2DATA) WHERE MS2PROD=(230.0924):TOLERANCEPPM=10:INTENSITYPERCENT=20 |
| Graminine | QUERY scaninfo(MS2DATA) WHERE MS2PROD=(178.0822 OR 160.0717):TOLERANCEPPM=10:INTENSITYPERCENT=20\|\|\|QUERY scaninfo(MS2DATA) WHERE MS2PROD=X AND MS2PROD=(X-177.0750):TOLERANCEPPM=10:INTENSITYPERCENT=20\|\|\| QUERY scaninfo(MS2DATA) WHERE MS2PROD=X AND MS2PROD=(X-159.0644):TOLERANCEPPM=10:INTENSITYPERCENT=20 |
| *N*-hydroxy-*N*-succinyl-cadaverine | QUERY scaninfo(MS2DATA) WHERE MS2PROD=(201.1234 OR 219.1339 OR 119.1179 OR 101.0233):TOLERANCEPPM=10:INTENSITYPERCENT=20 |
| *N*-acetyl-*N*-hydroxy-cadaverine | QUERY scaninfo(MS2DATA) WHERE MS2PROD=(161.1285):TOLERANCEPPM=10:INTENSITYPERCENT=20\|\|\|QUERY scaninfo(MS2DATA) WHERE MS2PROD=X AND MS2PROD=(X-160.1212):TOLERANCEPPM=10:INTENSITYPERCENT=20 |
| *N*-hydroxy-putrescine | QUERY scaninfo(MS2DATA) WHERE MS2PROD=(105.1022):TOLERANCEPPM=10:INTENSITYPERCENT=20\|\|\|QUERY scaninfo(MS2DATA) WHERE MS2PROD=X AND MS2PROD=(X-104.0950):TOLERANCEPPM=10:INTENSITYPERCENT=20 |
| *N*-hydroxy-*N*-succinyl-putrescine | QUERY scaninfo(MS2DATA) WHERE MS2PROD=(187.1077):TOLERANCEPPM=10:INTENSITYPERCENT=20\|\|\|QUERY scaninfo(MS2DATA) WHERE MS2PROD=X AND MS2PROD=(X-186.1004):TOLERANCEPPM=10:INTENSITYPERCENT=20 |
| *N*-acetyl-*N*-hydroxy-lysine | QUERY scaninfo(MS2DATA) WHERE MS2PROD=(205.1183 OR 187.1077):TOLERANCEPPM=10:INTENSITYPERCENT=20\|\|\|QUERY scaninfo(MS2DATA) WHERE MS2PROD=X AND MS2PROD=(X-186.1004):TOLERANCEPPM=10:INTENSITYPERCENT=20 |
| *N*-formyl-*N*-hydroxy-ornithine | QUERY scaninfo(MS2DATA) WHERE MS2PROD=(177.0870 OR 159.0764): TOLERANCEPPM=10:INTENSITYPERCENT=20\|\|\|QUERY scaninfo(MS2DATA) WHERE MS2PROD=X AND MS2PROD=(X-176.0797):TOLERANCEPPM=10:INTENSITYPERCENT=20\|\|\|QUERY scaninfo(MS2DATA) WHERE MS2PROD=X MS2PROD=(X-158.0691):TOLERANCEPPM=10:INTENSITYPERCENT=20 |
| *N*-acetyl-*N*-hydroxy-ornithine | QUERY scaninfo(MS2DATA) WHERE MS2PROD=(191.1026 OR 173.0921):TOLERANCEPPM=10:INTENSITYPERCENT=20\|\|\|QUERY scaninfo(MS2DATA) WHERE MS2PROD=X AND MS2PROD=(X-190.0954):TOLERANCEPPM=10:INTENSITYPERCENT=20\|\|\| QUERY scaninfo(MS2DATA) WHERE MS2PROD=X AND MS2PROD=(X-172.0848):TOLERANCEPPM=10:INTENSITYPERCENT=20 |
| Cyclo-*N*-hydroxy-ornithine | QUERY scaninfo(MS2DATA) WHERE MS2PROD=(131.0815):TOLERANCEPPM=10:INTENSITYPERCENT=20\|\|\|QUERY scaninfo(MS2DATA) WHERE MS2PROD=X AND MS2PROD=(X-130.0742):TOLERANCEPPM=10:INTENSITYPERCENT=20 |
| *N-*acetyl-*N*-hydroxy-aminopropane | QUERY scaninfo(MS2DATA) WHERE MS2PROD=(133.0972):TOLERANCEPPM=10:INTENSITYPERCENT=20\|\|\|QUERY scaninfo(MS2DATA) WHERE MS2PROD=X AND MS2PROD=(X-132.0899):TOLERANCEPPM=10:INTENSITYPERCENT=20 |
| *N*-anhydromevalonyl-*N*-hydroxy-ornithine | QUERY scaninfo(MS2DATA) WHERE MS2PROD=(261.1445):TOLERANCEPPM=10:INTENSITYPERCENT=20\|\|\|QUERY scaninfo(MS2DATA) WHERE MS2PROD=X AND MS2PROD=(X-260.1372):TOLERANCEPPM=10:INTENSITYPERCENT=20 |
| *N*-hydroxy-*N*-(3-hydroxybutyryl)-ornithine | QUERY scaninfo(MS2DATA) WHERE MS2PROD=(235.1288):TOLERANCEPPM=10:INTENSITYPERCENT=20\|\|\| QUERY scaninfo(MS2DATA) WHERE MS2PROD=X AND MS2PROD=(X-234.1216):TOLERANCEPPM=10:INTENSITYPERCENT=20 |
| *N*-hydroxy-histamine | QUERY scaninfo(MS2DATA) WHERE MS2PROD=(128.0818):TOLERANCEPPM=10:INTENSITYPERCENT=20 |
| Phenol oxazoline | QUERY scaninfo(MS2DATA) WHERE MS2PROD=(164.0706 OR 190.0499):TOLERANCEPPM=10:INTENSITYPERCENT=20 |
| Phenol methyloxazoline | QUERY scaninfo(MS2DATA) WHERE MS2PROD=(178.0863 OR 204.0655):TOLERANCEPPM=10:INTENSITYPERCENT=20 |
| Phenol thiazoline | QUERY scaninfo(MS2DATA) WHERE MS2PROD=(180.0478 OR 190.0321):TOLERANCEPPM=10:INTENSITYPERCENT=20 |
| Methylthiazolidine carboxylic acid | QUERY scaninfo(MS2DATA) WHERE MS2PROD=(146.0270):TOLERANCEPPM=10:INTENSITYPERCENT=20 |
| Methylthiazoline carboxylic acid | QUERY scaninfo(MS2DATA) WHERE MS2PROD=(144.0114 OR 160.0426):TOLERANCEPPM=10:INTENSITYPERCENT=20 |
| Catechol methyloxazoline | QUERY scaninfo(MS2DATA) WHERE MS2PROD=(220.0604 OR 194.0812):TOLERANCEPPM=10:INTENSITYPERCENT=20 |
| Catechol thiazoline | QUERY scaninfo(MS2DATA) WHERE MS2PROD=(222.0219 OR 194.0270):TOLERANCEPPM=10:INTENSITYPERCENT=20 |

**Table S3.** ^1^H (700 MHz) and ^13^C (175 MHz) NMR data of bisbulbichelin in DMSO-*d*_6_ (*J* in Hz, *δ* in ppm)

| No. | *δ*_C,_ type | *δ*_H,_ multiplicity (*J*, Hz) | HMBC |
| --- | --- | --- | --- |
| 1 | 159.0, C |  |  |
| 2 | 117.2, CH | 6.97, d (7.6) | C1, C4, C7 |
| 3 | 130.5, CH | 7.41, dd (7.6, 1.7) | C1 |
| 4 | 119.6, CH | 6.94, t (7.4) | C1, C2, C5 |
| 5 | 133.8, CH | 7.43, m | C1, C3, C7 |
| 6 | 116.23, C |  |  |
| 7 | 171.8, C |  |  |
| 8 | 35.9, CH_2_ | 3.56, m | C7, C9, C10 |
| 9 | 82.0, CH | 4.92, m | C7, C10 |
| 10 | 77.5, CH | 4.21, d (7.5) | C13 |
| 11 | 32.7, CH_2_ | 2.19, m |  |
| 12 | 74.8, CH | 3.45, m | C9, C10, C11, C17 |
| 13 | 43.3, CH_3_ | 2.48, s | C10, C12 |
| 14 | 75.9, CH | 4.25, d (5.6) | C11, C12 |
| 15 | 34.8, CH_2_ | 3.03, m | C14, C18 |
| 16 | 74.8, CH | 3.45, m | C17, C18 |
| 17 | 41.7, CH_3_ | 2.45, s |  |
| 18 | 170.8, C |  |  |
| 19 | 51.1, CH | 4.34, m | C20, C21 |
| 20 | 173.2, C |  |  |
| 21 | 31.02, CH_2_ | 2.94, m | C18 |
| 22 | 36.1, CH_2_ | 2.74, m | C19, C21 |
| NH |  | 8.08 | C18, C19, C21 |
| acid |  | 12.64 |  |

**Table S4.** ^1^H (800 MHz) and ^13^C (200 MHz) NMR data of Acyl petrobactin C10:0 in DMSO-*d*_6_ (*J* in Hz, *δ* in ppm)

| Group | No. | *δ*_C,_ type | *δ*_H,_ multiplicity (*J*, Hz) | HMBC |
| --- | --- | --- | --- | --- |
| 3,4-DHBA 1 | 1 | 126.1, C |  |  |
|  | 2 | 115.5, CH | 7.25, s | C3, C4, C6, C7 |
|  | 3 | 148.7, C |  |  |
|  | 4 | 144.9, C |  |  |
|  | 5 | 115.3, CH | 6.74, d (7.96) | C1 |
|  | 6 | 119.4, CH | 7.16, d (7.96) | C2, C3, C7 |
|  | 7 | 167.7, C |  |  |
| Spermidine 1 | 8 | 36.9, CH_2_ | 3.22, m |  |
|  | 9 | 29.2, CH_2_ | 1.73, m |  |
|  | 10 | 44.5, CH_2_ | 3.19, m | C35 |
|  | 11 | 42.8, CH_2_ | 3.24, m |  |
|  | 12 | 25.7, CH_2_ | 1.40, m |  |
|  | 13 | 25.8, CH_2_ | 1.48, m |  |
|  | 14 | 38.8, CH_2_ | 3.00, m |  |
| Citrate | 15 | 170.0, C |  |  |
|  | 16 | 43.8, CH_2_ | 2.59, 2.49, m | C15, C17, C18 |
|  | 17 | 74.1, C |  |  |
|  | 18 | 175.5, C |  |  |
|  | 19 | 43.6, CH_2_ | 2.57, 2.49, m | C17, C18, C20 |
|  | 20 | 169.7, C |  |  |
| Spermidine 2 | 21 | 38.0, CH_2_ | 3.06, m |  |
|  | 22 | 23.3, CH_2_ | 1.55, m |  |
|  | 23 | 25.8, CH_2_ | 1.43, m |  |
|  | 24 | 46.7, CH_2_ | 2.89, m |  |
|  | 25 | 45.1, CH_2_ | 2.90, m |  |
|  | 26 | 26.8, CH_2_ | 1.80, m |  |
|  | 27 | 36.6, CH_2_ | 3.27, m |  |
| 3,4-DHBA 2 | 28 | 167.2, C |  |  |
|  | 29 | 125.9, C |  |  |
|  | 30 | 115.5, CH | 7.27, s | C28, C31, C32, C34 |
|  | 31 | 148.7, C |  |  |
|  | 32 | 145.0, C |  |  |
|  | 33 | 115.3, CH | 6.77, d (7.96) | C29 |
|  | 34 | 119.4, CH | 7.18, d (7.96) | C28, C30, C31 |
| Acyl tail | 35 | 172.5, C |  |  |
|  | 36 | 32.5, CH_2_ | 2.26, 2.22, m | C35, C37, C38 |
|  | 37 | 29.5, CH_2_ | 1.71, m |  |
|  | 38 | 25.3, CH_2_ | 1.41, m |  |
|  | 39-43 | 22.5-31.6 | 1.47-1.21, m |  |
|  | 44 | 14.3, CH_3_ | 0.84, m |  |

**Table S5.** ^1^H (800 MHz) and ^13^C (200 MHz) NMR data of petrobactin in DMSO-*d*_6_ (*J* in Hz, *δ* in ppm)

| Group | No. | *δ*_C,_ type | *δ*_H,_ multiplicity (*J*, Hz) | HMBC | *δ*_C_, literature^40^ | *δ_H_*, literature^40^ |
| --- | --- | --- | --- | --- | --- | --- |
| 3,4-DHBA | 1 | 125.9, C |  |  | 125.36 |  |
|  | 2 | 115.5, CH | 7.26, s | C3, C4, C6, C7 | 115.04 | 7.28 |
|  | 3 | 148.7, C |  |  | 144.84 |  |
|  | 4 | 145.0, C |  |  | 148.45 |  |
|  | 5 | 115.3, CH | 6.77, d (6.97) | C1 | 114.79 | 6.76 |
|  | 6 | 119.4, CH | 7.18, d (6.97) | C2, C3, C7 | 118.93 | 7.18 |
|  | 7 | 167.2, C |  |  | 166.65 |  |
| Spermidine | 8 | 36.6, CH_2_ | 3.28, m | C10 | 36.13 | 3.28 |
|  | 9 | 26.5, CH_2_ | 1.80, m |  | 26.21 | 1..80 |
|  | 10 | 45.3, CH_2_ | 2.89, broad |  | 44.76 | 2.90 |
|  | 11 | 46.9, CH_2_ | 2.88, broad |  | 46.44 | 2.90 |
|  | 12 | 26.5, CH_2_ | 1.80, m | C10 | 26.00 | 1.42 |
|  | 13 | 23.5, CH_2_ | 1.55, m | C11 | 22.95 | 1.55 |
|  | 14 | 38.2, CH_2_ | 3.03, m | C13, C12 | 37.67 | 3.04 |
| Citrate | 15 | 170.2, C |  |  | 169.53 |  |
|  | 16 | 43.8, CH_2_ | 2.58, 2.49, d (2.57) | C15, C17, C18 | 43.3 | 2.59, 2.50 |
|  | 17 | 74.4, C |  |  | 73.45 |  |
|  | 18 | 175.8, C |  |  | 174.98 |  |

**Table S6.** Putative iron acquisition and transport-related proteins found in *Microbulbifer* sp. CNSA002

| **Function** | **Genomic coordinate** | **Predicted function** | **Tool** |
| --- | --- | --- | --- |
| Iron-binding proteins | 57,349-58,440 | Heme A synthase | NCBI PGAP^22^ |
|  | 58,478-59,371 | Heme O synthase | NCBI PGAP |
|  | 3,949,197-3,949,427 | Bacterioferritin-associated ferredoxin | NCBI PGAP |
|  | 3,950,573-3,951,064 | Bacterioferritin | NCBI PGAP |
| Membrane transporter | 65,905-66,705 | Iron chelate uptake ABC transporter family permease subunit | NCBI PGAP |
|  | 1,245,445-1,246,476 | Ferric iron ABC transporter, iron-binding protein | RAST-SEED^18-20^ |
|  | 1,249,333-1,248,290 | Ferric iron ABC transporter, ATP-binding protein | RAST-SEED |
|  | 1,246,598-1,248,283 | Ferric iron ABC transporter, permease protein | RAST-SEED |
|  | 1,950,272-1,952,443 | TonB-dependent hemoglobin/transferrin/lactoferrin family receptor | NCBI PGAP |
| Heme transporter | 1,743,731-1,744,444 | Heme-exporter protein CcmB | NCBI PGAP |
|  | 1,744,459-1,745,202 | Heme ABC transporter permease | NCBI PGAP |
|  | 1,745,204-1,745,446 | Heme-exporter protein CcmD | NCBI PGAP |
| Siderophore-iron uptake | 475,503-477,602 | Outer membrane receptor for ferric coprogen and ferric-rhodotorulic acid | RAST-SEED |
|  | 477,680-478,936 | Iron-uptake factor PiuB | RAST-SEED |
|  | 1,768,529-1,770,739 | TonB-dependent siderophore receptor | NCBI PGAP |
|  | 2,382,631-2,384,748 | TonB-dependent siderophore receptor | NCBI PGAP |
|  | 2,738,783-2,740,927 | TonB-dependent receptor | antiSMASH^21^ |
|  | 4,533,865-4,535,943 | TonB-dependent receptor | antiSMASH |
|  | 4,820,881-4,820,354 | Ferric siderophore transport system, biopolymer transport protein ExbB | RAST-SEED |
| NRPS-metallophore biosynthesis | 2,760,099-2,754,514 | Iron acquisition yersiniabactin synthesis enzyme (Irp2) | RAST-SEED |
|  | 2,768,167-2,766,752 | Isochorismate synthase of siderophore biosynthesis | antiSMASH, RAST-SEED |
|  | 2,768,493-2,768,164 | Isochorismate pyruvate-lyase, siderophore biosynthesis (pyochelin) | antiSMASH, RAST-SEED |
|  | 2,769,548-2,768,811 | Thioesterase in siderophore biosynthesis gene cluster | RAST-SEED |
|  | 2,769,670-2,771,274 | 2,3-dihydroxybenzoate-AMP ligase of siderophore biosynthesis | RAST-SEED |
| NI-siderophore biosynthesis, anthrachelin biosynthesis | 4,538,462-4,536,528 | Biosynthetic arginine decarboxylase | RAST-SEED |
|  | 4,541,802-4,543,643 | Anthrachelin biosynthesis protein AsbA | RAST-SEED |
|  | 4,543,630-4,545,549 | Anthrachelin biosynthesis protein AsbB | RAST-SEED |
|  | 4,545,533-4,546,840 | AMP-binding | antiSMASH |
|  | 4,546,884-4,548,260 | Phosphopantetheine (PP)-binding | antiSMASH |
|  | 4,548,257-4,549,105 | 3-dehydroshikimate dehydratase | BlastP^25^ |
|  | 4,549,159-4,550,046 | *N*-carbamoylputrescine amidase | BlastP |
|  | 4,551,139-4,550,105 | Agmatine deiminase | BlastP |

**Table S7.** *Microbulbifer* sp. CNSA002 NRPS siderophore biosynthetic gene cluster proteins predicted function

| Gene locus | Predicted function | Tool | Accession | Sorangibactin BGC homology (query cover%, identity%) | Anthrochelin BGC homology (query cover%, identity%) | Gene |
| --- | --- | --- | --- | --- | --- | --- |
| 2,738,000 - 2,738,683 | VOC family protein | BlastP | WP_413662051.1 | SrbJ (95,56) | AntJ (97, 54) | *bbchK* |
| 2,738,783 - 2,740,927 | TonB-dependent receptor | antiSMASH, BlastP | WP_413662052.1 |  | Unnamed, NII05597.1 (93, 48) | *bbchP* |
| 2,740,959 - 2,744,369 | ABC transporter permease | BlastP | WP_413662053.1 | SrbP (99, 32) | Unnamed, NII05598.1 (99, 43) | *bbchO* |
| 2,744,366 - 2,745,253 | ABC transporter ATP-binding protein | antiSMASH, BlastP | WP_413662054.1 | SrbO (100, 53) | Unnamed, NII05599.1 (98, 63) | *bbchN* |
| 2,745,330 - 2,746,436 | Gfo/Idh/MocA family oxidoreductase | BlastP |  | SrbI (99, 65) | AntI (97, 54) | *bbchJ* |
| 2,746,426 - 2,748,699 | Class I SAM-dependent methyltransferase | BlastP | WP_413662055.1 | SrbG (99, 38) | AntG (76, 37) | *bbchI* |
| 2,748,709 - 2,749,860 | Class I SAM-dependent methyltransferase | BastP | WP_413662056.1 | SrbF (81, 34) | AntF (91, 45) | *bbchH* |
| 2,749,857 - 2,750,957 | Saccharopine dehydrogenase NADP-binding domain-containing protein | BlastP | WP_413662057.1 | SrbE (89, 43) | AntE (99, 39) | *bbchG* |
| 2,750,954 - 2,752,708 | ABC transporter ATP-binding protein | antiSMASH, BlastP | WP_413662058.1 |  | AntD (99, 54) | *bbchM* |
| 2,752,705 - 2,754,513 | ABC transporter ATP-binding protein | antiSMASH, BlastP | WP_413662059.1 |  | AntC (94, 62) | *bbchL* |
| 2,754,514 - 2,760,099 | NRPS synthase | antiSMASH, BlastP | WP_413662060.1 | SrbD (100, 55) | AntB (100, 53) | *bbchF* |
| 2,760,148 - 2,766,774 | NRPS synthase | antiSMASH, BlastP | WP_413662061.1 | SrbC (99, 51) | AntA (97, 52) | *bbchE* |
| 2,766,752 - 2,768,167 | Isochorismate synthase | antiSMASH, BlastP | WP_413662062.1 | SrbB (54, 35) | AntN (98, 35) | *bbchD* |
| 2,768,164 - 2,768,493 | Isochorismate lyase | antiSMASH, BlastP | WP_413662063.1 |  | AntM (91, 48) | *bbchC* |
| 2,768,811 - 2,769,548 | Thioesterase | antiSMASH, BlastP | WP_413662064.1 |  | AntL (95, 39) | *bbchB* |
| 2,769,670 - 2,771,274 | AMP-binding | antiSMASH, BlastP | WP_413662065.1 | SrbK (99, 43) | AntK (98, 44) | *bbchA* |

**Table S8.** Petrobactin analogs detected in *Microbulbifer* sp. CNSA002 culture and datasets mined with MASST

| Name | Theoretical *m/z* [M+H]^+^ | Experimental *m/z* [M+H]^+^ | Error (ppm) | Acyl tail | Dataset | Extraction source |
| --- | --- | --- | --- | --- | --- | --- |
| Dehydroxypetrobactin | 703.366 | 703.366 | 0 | - | a | Sup. |
| Petrobactin | 719.361 | 719.361 | 0 | - | a | Sup. |
| Acyl dehydroxypetrobactin C2:0 | 745.377 | 745.376 | 1 | C2:0 | a | Sup. |
| Acyl dehydroxypetrobactin C3:0 | 759.392 | 759.393 | 1 | C3:0 | a | Sup. |
| Acyl petrobactin C2:0 | 761.372 | 761.371 | 1 | C2:0 | a | Sup. |
| Acyl petrobactin C3:0 | 775.387 | 775.387 | 0 | C3:0 | a | Sup. |
| Acyl petrobactin C7:0 | 831.450 | 831.450 | 0 | C7:0 | a, c | Sup. |
| Acyl dehydroxypetrobactin C9:0 | 843.486 | 843.487 | 1 | C9:0 | a | Sup. |
| Acyl petrobactin C8:0 | 845.466 | 845.466 | 0 | C8:0 | a, c | Sup. |
| Acyl didehydroxypetrobactin C11:0 | 855.523 | 855.522 | 1 | C11:0 | a | Sup. |
| Acyl dehydroxypetrobactin C10:0 | 857.502 | 857.500 | 2 | C10:0 | a | Sup. |
| Acyl petrobactin C9:0 | 859.481 | 859.481 | 0 | C9:0 | a, b, c | Sup., cell |
| Acyl didehydroxypetrobactin C12:1 | 867.523 | 867.522 | 1 | C12:1* | a | Sup. |
| Acyl dehydroxypetrobactin C11:1 | 869.502 | 869.504 | 2 | C11:1* | a | Sup., cell |
| Acyl petrobactin C10:1 | 871.481 | 871.481 | 0 | C10:1* | a, c | Sup. |
| Acyl dehydroxypetrobactin C11:0 | 871.518 | 871.518 | 0 | C11:0 | a | Sup., cell |
| Acyl petrobactin C10:0 | 873.497 | 873.497 | 0 | C10:0 | a, b, c | Sup., cell |
| Acyl dehydroxypetrobactin C12:1 | 883.518 | 883.518 | 0 | C12:1* | a | Sup., cell |
| Acyl petrobactin C11:1 | 885.497 | 885.495 | 2 | C11:1* | a, c | Sup. |
| Acyl dehydroxypetrobactin C12:0 | 885.533 | 885.533 | 0 | C12:0 | a | Sup., cell |
| Acyl petrobactin C11:0 | 887.512 | 887.511 | 1 | C11:0 | a, b, c | Sup., cell |
| Hydroxyacyl petrobactin C10:0 | 889.492 | 889.495 | 3 | C10:0-OH^Ⴕ^ | c | - |
| Acyl dehydroxypetrobactin C13:2 | 895.518 | 895.518 | 0 | C13:2* | a | Sup., cell |
| Acyl dehydroxypetrobactin C13:1 | 897.533 | 897.533 | 0 | C13:1* | a | Sup., cell |
| Acyl petrobactin C12:1 | 899.512 | 899.513 | 1 | C12:1* | a, b, c | Sup., cell |
| Acyl petrobactin C12:0 | 901.528 | 901.529 | 1 | C12:0 | a, b, c | Sup., cell |
| Acyl petrobactin C13:2 | 911.512 | 911.513 | 1 | C13:2* | a | Sup., cell |
| Acyl petrobactin C13:1 | 913.528 | 913.528 | 0 | C13:1* | a, b, c | Sup., cell |
| Hydroxyacyl petrobactin C12:1 | 915.507 | 915.513 | 6 | C12:1-OH* ^Ⴕ^ | c | - |
| Acyl petrobactin C13:0 | 915.544 | 915.543 | 1 | C13:0 | a, b, c | Sup., cell |
| Hydroxyacyl petrobactin C12:0 | 917.523 | 917.526 | 3 | C12:0-OH^Ⴕ^ | c | - |
| Acyl petrobactin C14:1 | 927.544 | 927.544 | 0 | C14:1* | a, b, c | Sup., cell |
| Hydroxyacyl petrobactin C13:1 | 929.523 | 929.522 | 1 | C13:1-OH* ^Ⴕ^ | a | Sup. |
| Acyl petrobactin C14:0 | 929.559 | 929.559 | 0 | C14:0 | a, b, c | Cell |
| Hydroxyacyl petrobactin C13:0 | 931.539 | 931.547 | 8 | C13:0-OH^Ⴕ^ | c | - |
| Acyl petrobactin C15:1 | 941.559 | 941.559 | 0 | C15:1* | a, b, c | Cell |
| Hydroxyacyl petrobactin C14:1 | 943.539 | 943.543 | 4 | C14:1-OH* ^Ⴕ^ | c | - |
| Acyl petrobactin C15:0 | 943.575 | 943.575 | 0 | C15:0 | a | Cell |
| Hydroxyacyl petrobactin C14:0 | 945.554 | 945.561 | 7 | C14:0-OH^Ⴕ^ | c | - |
| Acyl petrobactin C16:1 | 955.575 | 955.575 | 0 | C16:1* | a, b, c | Cell |
| Acyl petrobactin C16:0 | 957.591 | 957.590 | 1 | C16:0 | b, c | - |
| Acyl petrobactin C17:1 | 969.591 | 969.590 | 1 | C17:1* | a | Cell |
| Acyl petrobactin C17:0 | 971.606 | 971.605 | 1 | C17:0 | a | Cell |

* Position of the double bond was not determined

+ Position of the OH was not determined

^a^Detected in this work

^b^Detected in MSV000088745

^c^Detected in MSV000084873

**Table S9.** Phylogeny and coassociation analysis of IucA/IucC in *Microbulbifer* sp. CNSA002

| Organism | Accession number | Region (antiSMASH) | antiSMASH prediction | Similarity confidence |
| --- | --- | --- | --- | --- |
| *Bacterioplanoides* sp. SCSIO 12839 | NZ_CP073745.1 | 3,541,060 - 3,572,876 | Grimoviridin | Low |
| *Oleiphilus messinensi* | NZ_CP021425.1 | 567,559 - 599,336 | Roseobactin | Low |
| *Methylophaga thiooxydans* | NZ_JRQD00000000.1 | 78,582 - 110,098  (NZ_JRQD01000006.1) | Roseobactin | Low |
| *Saccharobesus litoralis* | NZ_CP026604.1 | 1,860,336 - 1,892,041 | NI-siderophore | - |
| *Litoribrevibacter albus* | NZ_BSNM00000000.1 | 482,023 - 514,043 (NZ_BSNM01000016.1) | NI-siderophore | - |
| *Microbulbifer* sp. A4B17 | NZ_CP029064.1 | 4,305,299 - 4,348,458 | NI-siderophore | - |
| *Roseimicrobium gellanilyticum* | NZ_QNRR00000000.1 | 1,173,095 - 1,212,091 (NZ_QNRR01000001.1) | NI-siderophore | - |
| *Alteromonas* sp. 76-1 | NZ_LR136958.1 | 3,765,400 - 3,881,208 | NI-siderophore | - |
| *Alteromonas* sp. KC14 | NZ_AP024236.1 | 2,189,584 - 2,221,334 | NI-siderophore | - |
| *Alteromonas* sp. Cnat2-8 | NZ_JAKREX000000000.1 | 123,821 - 155,517 (NZ_JAKREX010000004.1) | NI-siderophore | - |
| *Alteromonas* sp. BL110 | NZ_RAZV00000000.1 | 576,550 - 608,249 (NZ_RAZV01000010.1) | NI-siderophore | - |
| *Alteromonas portus* | NZ_JBMQEY000000000.1 | 1 - 30,183 (NZ_JBMQEY010000004.1) | NI-siderophore | - |

**Table S10.** *Microbulbifer* sp. CNSA002 NI-siderophore biosynthetic gene cluster proteins predicted function

| Gene locus | Predicted function | Tool | Accession | Petrobactin BGC homology (query cover%, identity%) | Gene |
| --- | --- | --- | --- | --- | --- |
| 4,533,865 - 4,535,943 | TonB-dependent receptor | antiSMASH, BlastP | WP_413663367.1 |  |  |
| 4,536,528 - 4,538,462 | Arginine decarboxylase | antiSMASH, BlastP | WP_413663368.1 |  | *asbG* |
| 4,538,473 - 4,538,802 | DUF2218 domain-containing protein | BlastP | WP_413663369.1 |  |  |
| 4,539,134 - 4,539,817 | Response regulator transcription factor | antiSMASH, BlastP | WP_413663370.1 |  |  |
| 4,540,294 - 4,541,676 | MATE efflux family protein | antiSMASH, BlastP | WP_413663371.1 |  |  |
| 4,541,802 - 4,543,643 | IucA/IucC family protein | antiSMASH, BlastP | WP_413663372.1 | *asbA* (98, 36) | *asbA* |
| 4,543,630 - 4,545,549 | IucA/IucC family protein | antiSMASH, BlastP | WP_413663373.1 | *asbB* (96, 32) | *asbB* |
| 4,545,533 - 4,546,840 | AMP-binding protein | antiSMASH, BlastP | WP_413663374.1 | *asbC* (100, 39) | *asbC* |
| 4,546,884 - 4,548,260 | PP-binding protein | antiSMASH | WP_413663375.1 | *asbD* (86, 28), *asbE* (96, 40) | *asbD*  *asbE* |
| 4,548,257 - 4,549,105 | 3-dehydroshikimate dehydratase | BlastP | WP_413663376.1 | *asbF* (94, 44) | *asbF* |
| 4,549,159 - 4,550,046 | *N*-carbamoylputrescine amidase | BlastP | WP_413663377.1 |  | *asbI* |
| 4,550,105 - 4,551,139 | Agmatine deiminase | BlastP | WP_413663378.1 |  | *asbH* |

**Table S11.** Theoretical m/z values for H-bound and all metal-bound acyl petrobactin and bulbichelin variants.

|  | **Theoretical *m/z* (z=1)** | | | | | |
| --- | --- | --- | --- | --- | --- | --- |
| **Name** | **H-bound** | **Fe-bound** | **Zn-bound** | **Co-bound** | **Ni-bound** | **Cu-bound** |
| Dehydroxypetrobactin | 703.3661 | 756.2770 | 765.2796 | 760.2836 | 759.2847 | 764.2790 |
| Petrobactin | 719.3610 | 772.2719 | 781.2745 | 776.2785 | 775.2796 | 780.2739 |
| Acyl dehydroxypetrobactin C2:0 | 745.3767 | 798.2876 | 807.2902 | 802.2942 | 801.2953 | 806.2896 |
| Acyl dehydroxypetrobactin C3:0 | 759.3923 | 812.3032 | 821.3058 | 816.3098 | 815.3109 | 820.3052 |
| Acyl petrobactin C2:0 | 761.3716 | 814.2825 | 823.2851 | 818.2891 | 817.2902 | 822.2845 |
| Acyl petrobactin C3:0 | 775.3872 | 828.2981 | 837.3007 | 832.3047 | 831.3058 | 836.3001 |
| Acyl petrobactin C7:0 | 831.4498 | 884.3607 | 893.3633 | 888.3673 | 887.3684 | 892.3627 |
| Acyl dehydroxypetrobactin C9:0 | 843.4862 | 896.3971 | 905.3997 | 900.4037 | 899.4048 | 904.3991 |
| Acyl petrobactin C8:0 | 845.4655 | 898.3764 | 907.3790 | 902.3830 | 901.3841 | 906.3784 |
| Acyl didehydroxypetrobactin C11:0 | 855.5226 | 908.4335 | 917.4361 | 912.4401 | 911.4412 | 916.4355 |
| Acyl dehydroxypetrobactin C10:0 | 857.5019 | 910.4128 | 919.4154 | 914.4194 | 913.4205 | 918.4148 |
| Acyl petrobactin C9:0 | 859.4811 | 912.3920 | 921.3946 | 916.3986 | 915.3997 | 920.3940 |
| Acyl didehydroxypetrobactin C12:1 | 867.5226 | 920.4335 | 929.4361 | 924.4401 | 923.4412 | 928.4355 |
| Acyl dehydroxypetrobactin C11:1 | 869.5019 | 922.4128 | 931.4154 | 926.4194 | 925.4205 | 930.4148 |
| Acyl petrobactin C10:1 | 871.4811 | 924.3920 | 933.3946 | 928.3986 | 927.3997 | 932.3940 |
| Acyl dehydroxypetrobactin C11:0 | 871.5175 | 924.4284 | 933.4310 | 928.4350 | 927.4361 | 932.4304 |
| Acyl petrobactin C10:0 | 873.4968 | 926.4077 | 935.4103 | 930.4143 | 929.4154 | 934.4097 |
| Acyl dehydroxypetrobactin C12:1 | 883.5175 | 936.4284 | 945.4310 | 940.4350 | 939.4361 | 944.4304 |
| Acyl petrobactin C11:1 | 885.4968 | 938.4077 | 947.4103 | 942.4143 | 941.4154 | 946.4097 |
| Acyl dehydroxypetrobactin C12:0 | 885.5332 | 938.4441 | 947.4467 | 942.4507 | 941.4518 | 946.4461 |
| Acyl petrobactin C11:0 | 887.5124 | 940.4233 | 949.4259 | 944.4299 | 943.4310 | 948.4253 |
| Hydroxyacyl petrobactin C10:0 | 889.4917 | 942.4026 | 951.4052 | 946.4092 | 945.4103 | 950.4046 |
| Acyl dehydroxypetrobactin C13:2 | 895.5175 | 948.4284 | 957.4310 | 952.4350 | 951.4361 | 956.4304 |
| Acyl dehydroxypetrobactin C13:1 | 897.5332 | 950.4441 | 959.4467 | 954.4507 | 953.4518 | 958.4461 |
| Acyl petrobactin C12:1 | 899.5124 | 952.4233 | 961.4259 | 956.4299 | 955.4310 | 960.4253 |
| Acyl petrobactin C12:0 | 901.5281 | 954.4390 | 963.4416 | 958.4456 | 957.4467 | 962.4410 |
| Acyl petrobactin C13:2 | 911.5124 | 964.4233 | 973.4259 | 968.4299 | 967.4310 | 972.4253 |
| Acyl petrobactin C13:1 | 913.5281 | 966.4390 | 975.4416 | 970.4456 | 969.4467 | 974.4410 |
| Hydroxyacyl petrobactin C12:1 | 915.5074 | 968.4183 | 977.4209 | 972.4249 | 971.4260 | 976.4203 |
| Acyl petrobactin C13:0 | 915.5437 | 968.4546 | 977.4572 | 972.4612 | 971.4623 | 976.4566 |
| Hydroxyacyl petrobactin C12:0 | 917.5230 | 970.4339 | 979.4365 | 974.4405 | 973.4416 | 978.4359 |
| Acyl petrobactin C14:1 | 927.5437 | 980.4546 | 989.4572 | 984.4612 | 983.4623 | 988.4566 |
| Hydroxyacyl petrobactin C13:1 | 929.5230 | 982.4339 | 991.4365 | 986.4405 | 985.4416 | 990.4359 |
| Acyl petrobactin C14:0 | 929.5594 | 982.4703 | 991.4729 | 986.4769 | 985.4780 | 990.4723 |
| Hydroxyacyl petrobactin C13:0 | 931.5387 | 984.4496 | 993.4522 | 988.4562 | 987.4573 | 992.4516 |
| Acyl petrobactin C15:1 | 941.5594 | 994.4703 | 1003.4729 | 998.4769 | 997.4780 | 1002.4723 |
| Hydroxyacyl petrobactin C14:1 | 943.5387 | 996.4496 | 1005.4522 | 1000.4562 | 999.4573 | 1004.4516 |
| Acyl petrobactin C15:0 | 943.5750 | 996.4859 | 1005.4885 | 1000.4925 | 999.4936 | 1004.4879 |
| Hydroxyacyl petrobactin C14:0 | 945.5543 | 998.4652 | 1007.4678 | 1002.4718 | 1001.4729 | 1006.4672 |
| Acyl petrobactin C16:1 | 955.5750 | 1008.4859 | 1017.4885 | 1012.4925 | 1011.4936 | 1016.4879 |
| Acyl petrobactin C16:0 | 957.5907 | 1010.5016 | 1019.5042 | 1014.5082 | 1013.5093 | 1018.5036 |
| Acyl petrobactin C17:1 | 969.5907 | 1022.5016 | 1031.5042 | 1026.5082 | 1025.5093 | 1030.5036 |
| Acyl petrobactin C17:0 | 971.6063 | 1024.5172 | 1033.5198 | 1028.5238 | 1027.5249 | 1032.5192 |
| Bulbichelin A | 525.1117 | 578.0226 | 587.0252 | 582.0292 | 581.0314 | 586.0256 |
| Bulbichelin B | 426.0974 | 479.0083 | 488.0109 | 483.0149 | 482.0171 | 487.0113 |
| Bulbichelin C | 543.1223 | 596.0332 | 605.0358 | 600.0398 | 599.042 | 604.0362 |

|  | **Theoretical *m/z* (z=2)** | | | | | |
| --- | --- | --- | --- | --- | --- | --- |
| **Name** | **H-bound** | **Fe-bound** | **Zn-bound** | **Co-bound** | **Ni-bound** | **Cu-bound** |
| Bulbichelin A | 263.0595 | 289.515 | 294.0162 | 291.5183 | 291.0193 | 293.5165 |
| Bulbichelin C | 272.0648 | 298.5202 | 303.0215 | 300.5235 | 300.0246 | 302.5217 |
| Bisbulbichelin | 542.1145 | 568.57 | 573.0712 | 570.5733 | 570.0743 | 572.5715 |

**Table S12.** Experimental m/z values for H-bound and all metal-bound acyl petrobactin and bulbichelin variants from direct-infusion untargeted LC-MS/MS.

|  | **Experimental *m/z* (z=1)** | | | | | |
| --- | --- | --- | --- | --- | --- | --- |
| **Name** | **H-bound** | **Fe-bound** | **Zn-bound** | **Co-bound** | **Ni-bound** | **Cu-bound** |
| Dehydroxypetrobactin | 703.3662 | 756.2790 | 765.2787 | 760.2855 | 759.2841 | 764.2815 |
| Petrobactin | 719.3612 | 772.2739 | 781.2750 | 776.2814 | ND | 780.2780 |
| Acyl dehydroxypetrobactin C2:0 | 745.3767 | ND | ND | ND | ND | ND |
| Acyl dehydroxypetrobactin C3:0 | ND | ND | ND | ND | ND | ND |
| Acyl petrobactin C2:0 | 761.3715 | 814.2849 | ND | 818.2881 | ND | 822.2841 |
| Acyl petrobactin C3:0 | 775.3862 | ND | ND | ND | ND | ND |
| Acyl petrobactin C7:0 | 831.4501 | ND | ND | ND | ND | ND |
| Acyl dehydroxypetrobactin C9:0 | 843.4889 | 896.4002 | ND | ND | ND | ND |
| Acyl petrobactin C8:0 | 845.4660 | 898.3787 | ND | ND | ND | ND |
| Acyl didehydroxypetrobactin C11:0 | 855.5229 | 908.4356 | ND | ND | ND | ND |
| Acyl dehydroxypetrobactin C10:0 | 857.5026 | 910.4141 | 919.4154 | 914.4148 | ND | 918.4135 |
| Acyl petrobactin C9:0 | 859.4820 | 912.3954 | 921.4010 | 916.4018 | ND | 920.3945 |
| Acyl didehydroxypetrobactin C12:1 | 867.5224 | ND | ND | ND | ND | ND |
| Acyl dehydroxypetrobactin C11:1 | 869.5020 | ND | 931.4188 | ND | ND | 930.4192 |
| Acyl petrobactin C10:1 | 871.4814 | 924.4004 | ND | ND | ND | 932.3973 |
| Acyl dehydroxypetrobactin C11:0 | 871.5171 | 924.4295 | 933.4235 | ND | ND | 932.4242 |
| Acyl petrobactin C10:0 | 873.4974 | 926.4105 | ND | 930.4192 | 929.4185 | ND |
| Acyl dehydroxypetrobactin C12:1 | 883.5172 | 936.4287 | 945.4327 | 940.4259 | 939.4403 | 944.4295 |
| Acyl petrobactin C11:1 | 885.4964 | 938.4131 | 947.4089 | ND | ND | ND |
| Acyl dehydroxypetrobactin C12:0 | 885.5334 | 938.4520 | ND | ND | ND | ND |
| Acyl petrobactin C11:0 | 887.5125 | 940.4259 | 949.4303 | 944.4295 | 943.4268 | 948.4326 |
| Hydroxyacyl petrobactin C10:0 | 889.4969 | ND | ND | ND | ND | ND |
| Acyl dehydroxypetrobactin C13:2 | 895.5188 | 948.4326 | 957.4388 | ND | 951.4469 | 956.4350 |
| Acyl dehydroxypetrobactin C13:1 | 897.5343 | 950.4436 | 959.4438 | 954.4413 | ND | 958.4424 |
| Acyl petrobactin C12:1 | 899.5124 | 952.4267 | ND | 956.4350 | ND | 960.4196 |
| Acyl petrobactin C12:0 | 901.5271 | 954.4404 | 963.4403 | 958.4424 | 957.4388 | 962.4372 |
| Acyl petrobactin C13:2 | 911.5124 | ND | 973.4268 | ND | ND | ND |
| Acyl petrobactin C13:1 | 913.5279 | 966.4414 | 975.4374 | 970.4531 | 969.4526 | 974.4341 |
| Hydroxyacyl petrobactin C12:1 | 915.5074 | ND | ND | ND | ND | ND |
| Acyl petrobactin C13:0 | 915.5419 | 968.4495 | ND | 972.4526 | ND | ND |
| Hydroxyacyl petrobactin C12:0 | 917.5233 | ND | ND | ND | ND | ND |
| Acyl petrobactin C14:1 | 927.5443 | 980.4634 | 989.4544 | 984.4555 | 983.4626 | 988.4508 |
| Hydroxyacyl petrobactin C13:1 | 929.5235 | ND | ND | ND | ND | ND |
| Acyl petrobactin C14:0 | 929.5551 | ND | ND | ND | ND | ND |
| Hydroxyacyl petrobactin C13:0 | 931.5379 | ND | ND | ND | ND | ND |
| Acyl petrobactin C15:1 | 941.5634 | ND | ND | ND | ND | ND |
| Hydroxyacyl petrobactin C14:1 | 943.5369 | ND | ND | ND | ND | ND |
| Acyl petrobactin C15:0 | 943.5789 | ND | ND | ND | ND | ND |
| Hydroxyacyl petrobactin C14:0 | ND | ND | ND | ND | ND | ND |
| Acyl petrobactin C16:1 | 955.5793 | ND | ND | ND | ND | ND |
| Acyl petrobactin C16:0 | ND | ND | ND | ND | ND | ND |
| Acyl petrobactin C17:1 | 969.5932 | ND | ND | ND | ND | ND |
| Acyl petrobactin C17:0 | ND | ND | ND | ND | ND | ND |
| Bulbichelin A | 525.1118 | 578.0248 | 587.0268 | 582.0276 | 581.0238 | 586.0256 |
| Bulbichelin B | 426.0972 | 479.0096 | 488.0135 | 483.0124 | 482.0152 | 487.0102 |
| Bulbichelin C | 543.1223 | ND | ND | ND | ND | 604.0292 |

|  | **Experimental *m/z* (z=2)** | | | | | |
| --- | --- | --- | --- | --- | --- | --- |
| **Name** | **H-bound** | **Fe-bound** | **Zn-bound** | **Co-bound** | **Ni-bound** | **Cu-bound** |
| Bulbichelin A | 263.0592 | 289.5182 | ND | 291.5189 | 291.0224 | ND |
| Bulbichelin C | ND | ND | ND | ND | ND | ND |
| Bisbulbichelin | 542.1142 | 568.5707 | 573.0717 | 570.5718 | 570.0711 | ND |

**Table S13.** Experimental rt values for H-bound and all metal-bound petrobactin and bulbichelin variants from direct infusion untargeted LC-MS/MS.

|  | **Retention time (min.) (z=1)** | | | | | |
| --- | --- | --- | --- | --- | --- | --- |
| **Name** | **H-bound** | **Fe-bound** | **Zn-bound** | **Co-bound** | **Ni-bound** | **Cu-bound** |
| Dehydroxypetrobactin | 1.81 | 1.80 | 1.80 | 1.79 | 1.83 | 1.89 |
| Petrobactin | 1.44 | 1.43 | 1.42 | 1.42 | ND | 1.42 |
| Acyl dehydroxypetrobactin C2:0 | 2.20 | ND | ND | ND | ND | ND |
| Acyl dehydroxypetrobactin C3:0 | ND | ND | ND | ND | ND | ND |
| Acyl petrobactin C2:0 | 2.14 | 2.14 | ND | 2.13 | ND | 2.22 |
| Acyl petrobactin C3:0 | 2.25 | ND | ND | ND | ND | ND |
| Acyl petrobactin C7:0 | 2.87 | ND | ND | ND | ND | ND |
| Acyl dehydroxypetrobactin C9:0 | 3.28 | 3.27 | ND | ND | ND | ND |
| Acyl petrobactin C8:0 | 3.05 | 3.05 | ND | ND | ND | ND |
| Acyl didehydroxypetrobactin C11:0 | 3.64 | 3.56 | ND | ND | ND | ND |
| Acyl dehydroxypetrobactin C10:0 | 3.47 | 3.42 | 3.52 | 3.45 | ND | 3.49 |
| Acyl petrobactin C9:0 | 3.21 | 3.21 | 3.27 | 3.23 | ND | 3.31 |
| Acyl didehydroxypetrobactin C12:1 | 3.67 | ND | ND | ND | ND | ND |
| Acyl dehydroxypetrobactin C11:1 | 3.56 | ND | 3.49 | ND | ND | 3.46 |
| Acyl petrobactin C10:1 | 3.41 | 3.40 | ND | ND | ND | 3.39 |
| Acyl dehydroxypetrobactin C11:0 | 3.60 | 3.60 | 3.58 | ND | ND | 3.51 |
| Acyl petrobactin C10:0 | 3.40 | 3.40 | ND | 3.46 | 3.43 | ND |
| Acyl dehydroxypetrobactin C12:1 | 3.62 | 3.61 | 3.59 | 3.56 | 3.70 | 3.55 |
| Acyl petrobactin C11:1 | 3.48 | 3.47 | 3.46 | ND | ND | ND |
| Acyl dehydroxypetrobactin C12:0 | 3.76 | 3.72 | ND | ND | ND | ND |
| Acyl petrobactin C11:0 | 3.53 | 3.56 | 3.60 | 3.55 | 3.57 | 3.60 |
| Hydroxyacyl petrobactin C10:0 | 3.01 | ND | ND | ND | ND | ND |
| Acyl dehydroxypetrobactin C13:2 | 3.69 | 3.60 | 3.65 | ND | 3.69 | 3.60 |
| Acyl dehydroxypetrobactin C13:1 | 3.73 | 3.68 | 3.69 | 3.86 | ND | 3.70 |
| Acyl petrobactin C12:1 | 3.54 | 3.54 | ND | 3.60 | ND | 3.58 |
| Acyl petrobactin C12:0 | 3.69 | 3.70 | 3.70 | 3.70 | 3.65 | 3.65 |
| Acyl petrobactin C13:2 | 3.68 | ND | 3.65 | ND | ND | ND |
| Acyl petrobactin C13:1 | 3.66 | 3.67 | 3.74 | 3.75 | 3.76 | 3.70 |
| Hydroxyacyl petrobactin C12:1 | 3.28 | ND | ND | ND | ND | ND |
| Acyl petrobactin C13:0 | 3.82 | 3.78 | ND | 3.77 | ND | ND |
| Hydroxyacyl petrobactin C12:0 | 3.46 | ND | ND | ND | ND | ND |
| Acyl petrobactin C14:1 | 3.82 | 3.83 | 3.85 | 3.80 | 3.89 | 3.82 |
| Hydroxyacyl petrobactin C13:1 | 3.62 | ND | ND | ND | ND | ND |
| Acyl petrobactin C14:0 | 3.83 | ND | ND | ND | ND | ND |
| Hydroxyacyl petrobactin C13:0 | 3.44 | ND | ND | ND | ND | ND |
| Acyl petrobactin C15:1 | 3.91 | ND | ND | ND | ND | ND |
| Hydroxyacyl petrobactin C14:1 | 3.62 | ND | ND | ND | ND | ND |
| Acyl petrobactin C15:0 | 4.07 | ND | ND | ND | ND | ND |
| Hydroxyacyl petrobactin C14:0 | ND | ND | ND | ND | ND | ND |
| Acyl petrobactin C16:1 | 4.07 | ND | ND | ND | ND | ND |
| Acyl petrobactin C16:0 | ND | ND | ND | ND | ND | ND |
| Acyl petrobactin C17:1 | 4.23 | ND | ND | ND | ND | ND |
| Acyl petrobactin C17:0 | ND | ND | ND | ND | ND | ND |
| Bulbichelin A | 3.91 | 3.93 | 3.92 | 3.93 | 3.93 | 3.93 |
| Bulbichelin B | 3.82 | 3.87 | 3.84 | 3.89 | 3.89 | 3.87 |
| Bulbichelin C | 4.83 | ND | ND | ND | ND | 4.77 |

|  | **Retention time (min.) (z=2)** | | | | | |
| --- | --- | --- | --- | --- | --- | --- |
| **Name** | **H-bound** | **Fe-bound** | **Zn-bound** | **Co-bound** | **Ni-bound** | **Cu-bound** |
| Bulbichelin A | 3.91 | 3.91 | ND | 3.94 | 3.94 | ND |
| Bulbichelin C | ND | ND | ND | ND | ND | ND |
| Bisbulbichelin | 5.97 | 5.99 | 6.01 | 5.96 | 6.10 | ND |

**Table S14.** Experimental H-bound peak area and % adduct values for all metal-bound petrobactin and bulbichelin variants from direct infusion untargeted LC-MS/MS.

|  |  | **% adduct (z=1)** | | | | |
| --- | --- | --- | --- | --- | --- | --- |
| **Name** | **Peak area H-bound** | **Fe-bound** | **Zn-bound** | **Co-bound** | **Ni-bound** | **Cu-bound** |
| Dehydroxypetrobactin | 1.26E+06 | 17 | 2 | 5 | 2 | 6 |
| Petrobactin | 8.17E+05 | 17 | 1 | 3 | ND | 5 |
| Acyl dehydroxypetrobactin C2:0 | 3.68E+04 | ND | ND | ND | ND | ND |
| Acyl dehydroxypetrobactin C3:0 | ND | ND | ND | ND | ND | ND |
| Acyl petrobactin C2:0 | 2.96E+05 | 18 | ND | 2 | ND | 1 |
| Acyl petrobactin C3:0 | 1.56E+04 | ND | ND | ND | ND | ND |
| Acyl petrobactin C7:0 | 3.21E+04 | ND | ND | ND | ND | ND |
| Acyl dehydroxypetrobactin C9:0 | 1.35E+05 | 70 | ND | ND | ND | ND |
| Acyl petrobactin C8:0 | 3.00E+05 | 36 | ND | ND | ND | ND |
| Acyl didehydroxypetrobactin C11:0 | 8.41E+04 | 49 | ND | ND | ND | ND |
| Acyl dehydroxypetrobactin C10:0 | 6.84E+05 | 30 | 0 | 2 | ND | 3 |
| Acyl petrobactin C9:0 | 5.61E+05 | 39 | 1 | 5 | ND | 5 |
| Acyl didehydroxypetrobactin C12:1 | 2.90E+05 | ND | ND | ND | ND | ND |
| Acyl dehydroxypetrobactin C11:1 | 2.17E+05 | ND | 0 | ND | ND | 1 |
| Acyl petrobactin C10:1 | 2.87E+05 | 32 | ND | ND | ND | 4 |
| Acyl dehydroxypetrobactin C11:0 | 9.40E+05 | 24 | 1 | ND | ND | 3 |
| Acyl petrobactin C10:0 | 2.34E+06 | 43 | ND | 6 | 2 | ND |
| Acyl dehydroxypetrobactin C12:1 | 2.38E+06 | 24 | 1 | 5 | 0 | 5 |
| Acyl petrobactin C11:1 | 5.75E+05 | 14 | 1 | ND | ND | ND |
| Acyl dehydroxypetrobactin C12:0 | 1.36E+06 | 32 | ND | ND | ND | ND |
| Acyl petrobactin C11:0 | 3.52E+06 | 40 | 4 | 6 | 2 | 6 |
| Hydroxyacyl petrobactin C10:0 | 3.36E+03 | ND | ND | ND | ND | ND |
| Acyl dehydroxypetrobactin C13:2 | 3.64E+05 | 3 | 0 | ND | 0 | 1 |
| Acyl dehydroxypetrobactin C13:1 | 2.17E+06 | 28 | 2 | 1 | ND | 5 |
| Acyl petrobactin C12:1 | 8.08E+06 | 40 | ND | 2 | ND | 2 |
| Acyl petrobactin C12:0 | 5.40E+06 | 44 | 1 | 6 | 1 | 2 |
| Acyl petrobactin C13:2 | 1.06E+06 | ND | 1 | ND | ND | ND |
| Acyl petrobactin C13:1 | 9.83E+06 | 39 | 3 | 6 | 2 | 6 |
| Hydroxyacyl petrobactin C12:1 | 5.31E+04 | ND | ND | ND | ND | ND |
| Acyl petrobactin C13:0 | 4.06E+06 | 49 | ND | 4 | ND | ND |
| Hydroxyacyl petrobactin C12:0 | 7.44E+03 | ND | ND | ND | ND | ND |
| Acyl petrobactin C14:1 | 5.41E+05 | 45 | 0 | 4 | 1 | 6 |
| Hydroxyacyl petrobactin C13:1 | 1.30E+05 | ND | ND | ND | ND | ND |
| Acyl petrobactin C14:0 | 2.92E+04 | ND | ND | ND | ND | ND |
| Hydroxyacyl petrobactin C13:0 | 2.33E+04 | ND | ND | ND | ND | ND |
| Acyl petrobactin C15:1 | 3.85E+04 | ND | ND | ND | ND | ND |
| Hydroxyacyl petrobactin C14:1 | 2.80E+04 | ND | ND | ND | ND | ND |
| Acyl petrobactin C15:0 | 1.93E+03 | ND | ND | ND | ND | ND |
| Hydroxyacyl petrobactin C14:0 | ND | ND | ND | ND | ND | ND |
| Acyl petrobactin C16:1 | 1.35E+04 | ND | ND | ND | ND | ND |
| Acyl petrobactin C16:0 | ND | ND | ND | ND | ND | ND |
| Acyl petrobactin C17:1 | 2.02E+04 | ND | ND | ND | ND | ND |
| Acyl petrobactin C17:0 | ND | ND | ND | ND | ND | ND |
| Bulbichelin A | 7.05E+06 | 61 | 95 | 82 | 16 | 100 |
| Bulbichelin B | 4.23E+05 | 13 | 38 | 35 | 2 | 100 |
| Bulbichelin C | 1.84E+05 | ND | ND | ND | ND | 100 |

|  |  | **% adduct (z=2)** | | | | |
| --- | --- | --- | --- | --- | --- | --- |
| **Name** | **Peak area H-bound** | **Fe-bound** | **Zn-bound** | **Co-bound** | **Ni-bound** | **Cu-bound** |
| Bulbichelin A | 2.57E+04 | 0 | ND | 99 | 30 | ND |
| Bulbichelin C | ND | ND | ND | ND | ND | ND |
| Bisbulbichelin | 1.17E+07 | 60 | 6 | 13 | 1 | ND |

SUPPLEMENTAL REFERENCES

(1) Monge-Loría, M.; Zhong, W.; Abrahamse, N. H.; Hartter, S.; Garg, N. Discovery of Peptidic Siderophore Degradation by Screening Natural Product Profiles in Marine-Derived Bacterial Mono- and Cocultures. *Biochemistry* **2025**, *64* (3), 634-654. DOI: 10.1021/acs.biochem.4c00706.

(2) Pluskal, T.; Castillo, S.; Villar-Briones, A.; Orešič, M. MZmine 2: Modular framework for processing, visualizing, and analyzing mass spectrometry-based molecular profile data. *BMC Bioinformatics* **2010**, *11* (1), 395. DOI: 10.1186/1471-2105-11-395.

(3) Mohimani, H.; Gurevich, A.; Mikheenko, A.; Garg, N.; Nothias, L.-F.; Ninomiya, A.; et al. Dereplication of peptidic natural products through database search of mass spectra. *Nature Chemical Biology* **2017**, *13* (1), 30-37. DOI: 10.1038/nchembio.2219.

(4) Mohimani, H.; Gurevich, A.; Shlemov, A.; Mikheenko, A.; Korobeynikov, A.; Cao, L.; et al. Dereplication of microbial metabolites through database search of mass spectra. *Nature Communications* **2018**, *9* (1), 4035. DOI: 10.1038/s41467-018-06082-8.

(5) Cao, L.; Guler, M.; Tagirdzhanov, A.; Lee, Y.-Y.; Gurevich, A.; Mohimani, H. MolDiscovery: Learning Mass Spectrometry Fragmentation of Small Molecules. *Nature Communications* **2021**, *12* (1), 3718. DOI: DOI: 10.1038/s41467-021-23986-0.

(6) van der Hooft, J. J. J.; Wandy, J.; Barrett, M. P.; Burgess, K. E. V.; Rogers, S. Topic modeling for untargeted substructure exploration in metabolomics. *Proceedings of the National Academy of Sciences* **2016**, *113* (48), 13738-13743. DOI: doi:10.1073/pnas.1608041113.

(7) Dührkop, K.; Fleischauer, M.; Ludwig, M.; Aksenov, A. A.; Melnik, A. V. SIRIUS 4: A Rapid Tool for Turning Tandem Mass Spectra into Metabolite Structure Information. *Nature Methods* **2019**, *16*, 299-302. DOI: DOI: 10.1038/s41592-019-0344-8.

(8) Dührkop, K.; Shen, H.; Meusel, M.; Rousu, J.; S., B. Searching Molecular Structure Databases with Tandem Mass Spectra Using CSI:FingerID. *Proceedings of the National Academy of Sciences* **2015**, *112* (41), 12580-12585. DOI: DOI: 10.1073/pnas.1509788112.

(9) Dührkop, K.; Nothias, L.-F.; Fleischauer, M.; Reher, R.; Ludwig, M.; Hoffmann, M. A.; et al. Systematic Classification of Unknown Metabolites Using High-Resolution Fragmentation Mass Spectra. *Nature Biotechnology* **2020**, *39*, 462-471. DOI: DOI: 10.1038/s41587-020-0740-8.

(10) van Santen, J. A.; Jacob, G.; Singh, A. L.; Aniebok, V.; Balunas, M. J.; Bunsko, D.; et al. The Natural Products Atlas: An Open Access Knowledge Base for Microbial Natural Products Discovery. *ACS Central Science* **2019**, *5* (11), 1824-1833. DOI: 10.1021/acscentsci.9b00806.

(11) Rutz, A.; Sorokina, M.; Galgonek, J.; Mietchen, D.; Willighagen, E.; Gaudry, A.; et al. The LOTUS initiative for open knowledge management in natural products research. *Elife* **2022**, *11*. DOI: 10.7554/eLife.70780.

(12) MarinLit: A Database of the Marine Natural Products Literature. Royal Society of Chemistry. <http://pubs.rsc.org/marinlit/> (accessed August 10 2023).

(13) Shannon, P.; Markie, A.; Ozier, O.; Baliga, N. S.; Wang, J. T.; Ramage, D.; et al. Cytoscape: A Software Environment for Integrated Models of Biomolecular Interaction Networks. *Genome Research* **2003**, *13*, 2498-2504. DOI: 10.1101/gr.1239303.

(14) Wishart, D. S.; Sayeeda, Z.; Budinski, Z.; Guo, A.; Lee, B. L.; Berjanskii, M.; et al. NP-MRD: the Natural Products Magnetic Resonance Database. *Nucleic Acids Res* **2022**, *50* (D1), D665-d677. DOI: 10.1093/nar/gkab1052.

(15) Pérez-Miranda, S.; Cabirol, N.; George-Téllez, R.; Zamudio-Rivera, L. S.; Fernández, F. J. O-CAS, a fast and universal method for siderophore detection. *Journal of Microbiological Methods* **2007**, *70* (1), 127-131. DOI: 10.1016/j.mimet.2007.03.023.

(16) Guan, L. L.; Onuki, H.; Kamino, K. Bacterial Growth Stimulation with Exogenous Siderophore and Synthetic *N*-Acyl Homoserine Lactone Autoinducers under Iron-Limited and Low-Nutrient Conditions. *Applied and Environmental Microbiology* **2000**, *66* (7), 2797-2803. DOI: doi:10.1128/AEM.66.7.2797-2803.2000.

(17) Rodriguez, R. L.; Gunturu, S.; Harvey, W. T.; Rosselló-Mora, R.; Tiedje, J. M.; Cole, J. R.; et al. The Microbial Genomes Atlas (MiGA) webserver: taxonomic and gene diversity analysis of Archaea and Bacteria at the whole genome level. *Nucleic Acids Res* **2018**, *46* (W1), W282-w288. DOI: 10.1093/nar/gky467.

(18) Aziz, R. K.; Bartels, D.; Best, A. A.; DeJongh, M.; Disz, T.; Edwards, R. A.; et al. The RAST Server: rapid annotations using subsystems technology. *BMC Genomics* **2008**, *9*, 75. DOI: 10.1186/1471-2164-9-75.

(19) Brettin, T.; Davis, J. J.; Disz, T.; Edwards, R. A.; Gerdes, S.; Olsen, G. J.; et al. RASTtk: A modular and extensible implementation of the RAST algorithm for building custom annotation pipelines and annotating batches of genomes. *Scientific Reports* **2015**, *5* (1), 8365. DOI: 10.1038/srep08365.

(20) Overbeek, R.; Olson, R.; Pusch, G. D.; Olsen, G. J.; Davis, J. J.; Disz, T.; et al. The SEED and the Rapid Annotation of microbial genomes using Subsystems Technology (RAST). *Nucleic Acids Research* **2013**, *42* (D1), D206-D214. DOI: 10.1093/nar/gkt1226.

(21) Blin, K.; Shaw, S.; Augustijn, H. E.; Reitz, Z. L.; Biermann, F.; Alanjary, M.; et al. antiSMASH 7.0: new and improved predictions for detection, regulation, chemical structures and visualisation. *Nucleic Acids Research* **2023**, *51* (W1), W46-W50. DOI: 10.1093/nar/gkad344.

(22) Tatusova, T.; DiCuccio, M.; Badretdin, A.; Chetvernin, V.; Nawrocki, E. P.; Zaslavsky, L.; et al. NCBI prokaryotic genome annotation pipeline. *Nucleic Acids Res* **2016**, *44* (14), 6614-6624. DOI: 10.1093/nar/gkw569.

(23) van den Belt, M.; Gilchrist, C.; Booth, T. J.; Chooi, Y.-H.; Medema, M. H.; Alanjary, M. CAGECAT: The CompArative GEne Cluster Analysis Toolbox for rapid search and visualisation of homologous gene clusters. *BMC Bioinformatics* **2023**, *24* (1), 181. DOI: 10.1186/s12859-023-05311-2.

(24) Zdouc, Mitja M.; Blin, K.; Louwen, Nico L. L.; Navarro, J.; Loureiro, C.; Bader, Chantal D.; et al. MIBiG 4.0: advancing biosynthetic gene cluster curation through global collaboration. *Nucleic Acids Research* **2024**, *53* (D1), D678-D690. DOI: 10.1093/nar/gkae1115.

(25) Camacho, C.; Coulouris, G.; Avagyan, V.; Ma, N.; Papadopoulos, J.; Bealer, K.; et al. BLAST+: architecture and applications. *BMC Bioinformatics* **2009**, *10*, 421. DOI: 10.1186/1471-2105-10-421.

(26) Larkin, M. A.; Blackshields, G.; Brown, N. P.; Chenna, R.; McGettigan, P. A.; McWilliam, H.; et al. Clustal W and Clustal X version 2.0. *Bioinformatics* **2007**, *23* (21), 2947-2948. DOI: 10.1093/bioinformatics/btm404.

(27) Tamura, K.; Stecher, G.; Kumar, S. MEGA11: Molecular Evolutionary Genetics Analysis Version 11. *Mol Biol Evol* **2021**, *38* (7), 3022-3027. DOI: 10.1093/molbev/msab120.

(28) Jones, D. T.; Taylor, W. R.; Thornton, J. M. The rapid generation of mutation data matrices from protein sequences. *Comput Appl Biosci* **1992**, *8* (3), 275-282. DOI: 10.1093/bioinformatics/8.3.275.

(29) Letunic, I.; Bork, P. Interactive Tree of Life (iTOL) v6: recent updates to the phylogenetic tree display and annotation tool. *Nucleic Acids Research* **2024**, *52* (W1), W78-W82. DOI: 10.1093/nar/gkae268.

(30) Edgar, R. C. MUSCLE: multiple sequence alignment with high accuracy and high throughput. *Nucleic Acids Res* **2004**, *32* (5), 1792-1797. DOI: 10.1093/nar/gkh340.

(31) Caswell, B. T.; de Carvalho, C. C.; Nguyen, H.; Roy, M.; Nguyen, T.; Cantu, D. C. Thioesterase enzyme families: Functions, structures, and mechanisms. *Protein Sci* **2022**, *31* (3), 652-676. DOI: 10.1002/pro.4263.

(32) Zallot, R.; Oberg, N.; Gerlt, J. A. The EFI Web Resource for Genomic Enzymology Tools: Leveraging Protein, Genome, and Metagenome Databases to Discover Novel Enzymes and Metabolic Pathways. *Biochemistry* **2019**, *58* (41), 4169-4182. DOI: 10.1021/acs.biochem.9b00735.

(33) Oberg, N.; Zallot, R.; Gerlt, J. A. EFI-EST, EFI-GNT, and EFI-CGFP: Enzyme Function Initiative (EFI) Web Resource for Genomic Enzymology Tools. *Journal of Molecular Biology* **2023**, *435* (14), 168018. DOI: 10.1016/j.jmb.2023.168018.

(34) Consortium, T. U. UniProt: the Universal Protein Knowledgebase in 2023. *Nucleic Acids Research* **2022**, *51* (D1), D523-D531. DOI: 10.1093/nar/gkac1052 (acccessed 9/3/2024).

(35) Miller, M. A.; Schwartz, T.; Pickett, B. E.; He, S.; Klem, E. B.; Scheuermann, R. H.; et al. A RESTful API for Access to Phylogenetic Tools via the CIPRES Science Gateway. *Evolutionary Bioinformatics* **2015**, *11* (4726-EBO-A-RESTful-API-for-Access-to-Phylogenetic-Tools-via-the-CIPRES-Science-.pdf), 43-48. DOI: 10.4137/EBO.S21501.

(36) Stamatakis, A. RAxML version 8: a tool for phylogenetic analysis and post-analysis of large phylogenies. *Bioinformatics* **2014**, *30* (9), 1312-1313. DOI: 10.1093/bioinformatics/btu033.

(37) Aron, A. T.; Petras, D.; Schmid, R.; Gauglitz, J. M.; Büttel, I.; Antelo, L.; et al. Native mass spectrometry-based metabolomics identifies metal-binding compounds. *Nat Chem* **2022**, *14* (1), 100-109. DOI: 10.1038/s41557-021-00803-1.

(38) Loos, M.; Gerber, C.; Corona, F.; Hollender, J.; Singer, H. Accelerated Isotope Fine Structure Calculation Using Pruned Transition Trees. *Analytical Chemistry* **2015**, *87* (11), 5738-5744. DOI: 10.1021/acs.analchem.5b00941.

(39) Damiani, T.; Jarmusch, A. K.; Aron, A. T.; Petras, D.; Phelan, V. V.; Zhao, H. N.; et al. A universal language for finding mass spectrometry data patterns. *Nature Methods* **2025**. DOI: 10.1038/s41592-025-02660-z.

(40) Bergeron, R. J.; Huang, G.; Smith, R. E.; Bharti, N.; McManis, J. S.; Butler, A. Total synthesis and structure revision of petrobactin. *Tetrahedron* **2003**, *59* (11), 2007-2014. DOI: 10.1016/S0040-4020(03)00103-0.
